# Associations between osteoarthritis and comorbidities before and after diagnosis across four European primary care databases: a multinational case–control and cohort study

**DOI:** 10.1016/j.lanepe.2026.101800

**Published:** 2026-08-11

**Authors:** Subhashisa Swain, Arani Vivekanantham, Andrea Dell ‘Isola, Anne Kamps, Marta Pineda Moncusi, Jos Runhaar, Chang Fu Kuo, Stevie Vanhegan, Irene Pistillidou, Jenny Cockshull, Christian Mallen, Michael Doherty, Daniel Prieto-Alhambra, Martin Englund, Sita Bierma-Zeinstra, Carol Coupland, Weiya Zhang

**Affiliations:** a Academic Rheumatology, School of Medicine, University of Nottingham, UK; b School of Medicine, Keele University, UK; c Nuffield Department of Primary Care Health Sciences, University of Oxford, UK; d Centre for Statistics in Medicine, NDORMS, University of Oxford, UK; e Clinical Epidemiology Unit, Orthopedics, Department of Clinical Sciences SHR, SHR University, Sweden; f Department of General Practice, Erasmus MC University Medical Center Rotterdam, the Netherlands; g Division of Rheumatology, Allergy and Immunology, Chang Gung Memorial Hospital, Taiwan; h Public and Patient Representatives from Cyprus, UK, and the Netherlands; i Pain Centre Versus Arthritis, University of Nottingham, UK; j Department of General Practice, Department of Orthopedic Surgery & Sports Medicine, Erasmus MC University Medical Center Rotterdam, the Netherlands; k Centre for Academic Primary Care, School of Medicine, University of Nottingham, UK

**Keywords:** Chronic disease, Osteoarthritis, Primary care, Europe, Multimorbidity, Epidemiology, Association

## Abstract

**Background:**

Osteoarthritis (OA) is associated with some comorbidities. However, the consistency of the associations across different countries is unclear. We analysed relationships between OA and 61 comorbidities before and after diagnosis using data from four European primary care databases: the UK, the Netherlands, Sweden, and Spain.

**Methods:**

The multinational ComOA study combined case–control and cohort designs in people aged ≥18 years using large primary care records databases in each country. The study population consisted of incident OA cases, and age and sex matched controls without OA. We reported adjusted odds ratios (aOR) and adjusted hazard ratios (aHR) after adjusting for other covariates. Pooled proportions with comorbidities, ORs and HRs were estimated using random effect methods for congruency evidence. Congruency was present if the results of all the countries favoured in one direction.

**Findings:**

The four databases included 845,373 OA cases and 2,556,243 matched controls combined for prospective and retrospective studies. The percentage of women varied from 41.7% (808,725/1,936,792) in Spain to 64.0% (254,930/398,143) in the Netherlands. Mean age at diagnosis of OA was lowest in the UK (59.8 years, SD 12.8), and highest in the Netherlands (65.8 years, SD 12.2). Retrospectively, 10 out of 33 comorbidities studied by all four countries showed congruent associations with OA such as, fibromyalgia (aOR 1.93; 95% CI 1.49–2.49), polymyalgia rheumatica (aOR 1.44; 95% CI 1.31–1.58), and chronic back pain (aOR 1.42; 95% CI 1.32–1.53). Prospectively, three of eight conditions studied by all four countries showed congruent, i.e., depression (aHR 1.11; 95% CI 1.07–1.16), osteoporosis (aHR 1.05; 95% CI 1.01–1.09), and hypertension (aHR 1.05; 95% CI 1.02–1.09). Additionally, contingency was also observed between retrospective and prospective studies for sleep problem, migraine, asthma, eczema, and benign prostatic hypertrophy.

**Interpretation:**

OA is associated with some long-term conditions across Europe, despite different population structures and health systems. Further research is needed to understand the mechanisms shared between OA and comorbidities for these associations, especially those congruent across nations.

**Funding:**

This work was supported by 10.13039/501100014034Foundation for Research in Rheumatology (FOREUM) grant The 10.13039/501100004359Swedish Research Council, Governmental funding of clinical research within the national health services (ALF), and The Swedish Rheumatism Association. CM is funded by the 10.13039/501100000272National Institute for Health Research (NIHR) Applied Research Collaboration West Midlands, the 10.13039/501100000272National Institute for Health Research (NIHR) School for Primary Care Research and a 10.13039/501100000272National Institute for Health Research (NIHR) Research Professorship in General Practice.

**Research in context**

**Evidence before this study**

Osteoarthritis (OA), a common chronic joint condition affecting millions globally, is increasingly seen as part of a larger health picture involving other chronic conditions. In our previously published systematic review, we did not find any articles comparing the result across the countries.

We performed an updated search in PubMed from inception to Jan 1, 2025, using following terms- ((“european people” [MeSH Terms] OR (“european” [All Fields] AND “people” [All Fields]) OR “european people” [All Fields] OR “european” [All Fields] OR “europeans” [All Fields]) AND (“countries” [All Fields] OR “country” [All Fields] OR “country s” [All Fields] OR “countrys” [All Fields]) AND (“osteoarthritis” [MeSH Terms] OR “osteoarthritis” [All Fields] OR “osteoarthritides” [All Fields]) AND (“comorbid” [All Fields] OR “comorbidity” [MeSH Terms] OR “comorbidity” [All Fields] OR “comorbidities” [All Fields] OR “comorbids” [All Fields])) OR (“multimorbid” [All Fields] OR “multimorbidity” [MeSH Terms] OR “multimorbidity” [All Fields] OR “multimorbidities” [All Fields]). We included observational and experimental studies on comorbidities in OA comparing the results from different nations. The search yielded 14,502 articles. A few articles (<9) studies the comorbidities in their respective databases. However, we could not find any articles providing comparison between countries.

This study addresses this critical gap by examining the bidirectional relationships between OA and 61 comorbidities across four European countries with distinct healthcare systems–United Kingdom, the Netherlands, Sweden, and Spain–using comprehensive primary care data.

**Added value of this study**

This is the first large-scale, multicentre study to assess the congruency of OA associated comorbidities across Europe, drawing on data from over 845,000 individuals with OA and 2.5 million matched controls. It confirms previously reported associations with musculoskeletal, cardiovascular and psychological conditions such as fibromyalgia (nearly twice as common), polymyalgia rheumatica, chronic back pain, depression, osteoporosis, and hypertension. Importantly, the study also identified new, consistently observed associations across countries, including allergy/eczema, migraine, vertigo, hearing impairment, and benign prostatic hypertrophy that warrant further investigation. The remarkable consistency of these findings across diverse populations suggests that these patterns reflect universal biological and clinical links between OA and other chronic conditions despite variations in population demographics and healthcare structures.

**Implications of all the available evidence**

The study confirms that OA is more than a localised joint disease; it is intricately linked to other long-term conditions Further research is needed to investigate shared risk factors and direct or indirect biological mechanisms. These findings enhance our understanding of OA's impact on overall health as well as offer a robust foundation for future research to uncover the mechanisms underpinning these associations, ultimately informing prevention and treatment strategies worldwide. The consistent associations across countries advocate for a holistic approach to OA management, emphasising early identification and integrated care strategies that address both OA and its related comorbidities. Such efforts could lead to better prevention and treatments, improving outcomes for people with OA in Europe and worldwide.

## Introduction

Osteoarthritis (OA) is one of the most common long-term conditions in the world. Globally, 595 million people had OA in 2020, equal to 7·6% of the global population.^1^ OA affects synovial joints such as knees, hips, hands and feet in adults.^2^ It is by far the most common form of arthritis, and a foremost cause of chronic pain and disability in older people.^3^^,^^4^ It is estimated that the burden of OA will continue to rise in the coming decades because of population ageing and the increasing prevalence of obesity: two major risk factors for OA.^5^

Comorbidity in OA is common, especially the presence of stroke, peptic ulcer, hypertension, and depression.^6^^,^^7^ This can occur due to multiple factors ranging from shared risk factors to exposure to medications.^8^ However, the OA comorbidity research to date is restricted to smaller number of conditions.^9^ Also there has been wide heterogeneity between studies in terms of the definitions of OA and other chronic conditions, the diagnosis and recording of diseases, sample sizes, and the number of diseases studied.^6^

Primary care is the usual setting for the management of people with OA. Presence of multiple conditions increases the complexity of the management and care needs of an individual.^10^ However, additional multiple long-term conditions (MLTCs) in OA can make treatment choices difficult. There is little evidence available concerning the association of MLTCs beyond psychological, cardiovascular, and musculoskeletal conditions in people with OA. Furthermore, it is difficult to compare results from different countries because of diversity in health systems, practices, and policies, and the knowledge of general practitioners. Also having a consensus on both the count and type of conditions with uniform methods and definitions in computing these estimates would reduce heterogeneity and make the comparison across countries more reliable.

To date, few studies have utilised large electronic records to explore the comorbidities of OA across countries. We aimed to compare and contrast the association of comorbidities in people with OA using national primary care registration databases from four European countries, specifically the UK, the Netherlands, Sweden, and Spain.

## Methods

We used the study population defined in a previous ComOA (Comorbidities in OA) consortium,^11^ that described in detail the associations of the comorbidities in people with OA. In this study, we compiled results from the four different countries on the prevalence and incidence of comorbidities in people with OA versus people without OA.

### Databases

Four routinely collected health databases were used in this study, two were national (the UK CPRD and the Netherlands IPCI) and two were regional (the Swedish SHR and Spanish SIDIAP)).^12^, ^13^, ^14^, ^15^ The four databases contain information about the population with primary care consultations in their respective areas and Swedish SHR had additional information from specialist care. The details about the database and their properties on coverage, completeness, validation, and reproducibility can be found elsewhere.^11^

In the UK, the Clinical Practice Research Data-link (CPRD) contribution was approved by the Independent Scientific Advisory Council (ISAC) 19/30R. In the Netherlands and Spain, the Integrated Primary Care Information (IPCI) registration no. 11/2019 and the Information System for the Development of Research in Primary Care (SIDIAP) 4R19/011 approved it, respectively. In Sweden, the Ethical Review Authority, Skåne Healthcare Register (SHR), ‘Dnr 2011-432, Dnr 2014-276, and Dnr 2018-233, approved it. Each registry database made available anonymised patient records for the research purposes. Individual informed consent was not required.

### Study design

The study centres used a combined retrospective case–control and prospective cohort study design for assessing associations with comorbidities recorded prior to and after the first recording of diagnosis of OA, respectively. In the retrospective study, OA cases and sex, age (±2 years), first year of registration, and practice matched or time varying exposure controls (1:1–4) without OA were used to determine the prevalence, incidence of comorbidities in OA. Both the groups were retrospectively reviewed for prior diagnoses of comorbidities and prospectively followed up for new comorbidities. For the prospective analysis, participants with incident OA but without the specific comorbidity of interest at the index date (i.e., people at risk) and matched controls without OA were followed up until the date of the first diagnosis of the comorbidity, deregistration, or death whichever comes first. Details of the study design can be accessed elsewhere.^11^

### Study duration and participants

Each study centre had data available for different lengths of observation.^11^ We decided to have a minimum follow-up of five years and centres with longer registration periods used the entire length of data available. People eligible for the study were those registered with the respective databases, were aged 18 years or above, flagged as acceptable data, and a minimum of one-year registration period available in the database.

### Definition of osteoarthritis

We identified people with a physician diagnosis of OA for the hip, knee, ankle or foot, wrist or hand, or site recorded as ‘unspecified’ during the study period. We used data specific coding systems such as the Read Codes for CPRD UK, the International Classification of Primary Care (ICPC-2) for the Netherlands and the ICD-10 for the Sweden and Spain databases. People with any previous recording of OA prior to the start date of the study were excluded.

Each person with OA was matched to controls without OA. Study centres used different approaches to select matched controls based on the feasibility of the databases. Using Incidence density sampling, up to four controls were matched to each OA case by age (±2 years), sex, first year of registration, and practice. Each control was assigned the same index date (date of first recorded OA diagnosis) as their matched case, apart from the time varying exposure control.

### Comorbidities

We defined comorbidity as the recording of a diagnosis of predefined chronic conditions in individuals using either ICD-10 or Read or ICPC-2 codes depending on the database. An extensive list of 61 chronic conditions was prepared from the Quality Outcome Framework (QOF),^16^ the list of the US Department of Health and Human Services Initiative on Multiple Chronic Conditions,^17^ the Global Burden of Diseases^18^ and the Charlson comorbidity index.^19^ The list had been updated with findings from our systematic review^6^ and a previous UK community-based knee pain study^6^^,^^20^ by including common and important comorbidities not included in the above.^21^^,^^22^ The detailed list of the conditions is given in Supplementary Tables S1 and S2.

### Covariates

Because of different information available across the countries, we agreed to use the maximum available covariates in each of the respective databases. The list of relevant available covariates was age, sex, body mass index (BMI), smoking, alcohol use, socioeconomic variables such as education level, income and place of birth (to identify those who immigrated to the country), residential area, and marital status (or registered partner). All centres had age, and sex as common covariates.

### Data harmonisation

We followed a code mapping exercise for identification of people with OA and other comorbidities. Details of the method is described elsewhere.^11^ Where possible we used a maximum number of chronic conditions available from list of 61 conditions and covariates common to all the databases.

### Outcomes

Firstly, we estimated the prevalence of a specific comorbidity at the index date and its association with OA diagnosed at the index date (the date of the first diagnosis of OA). Then we calculated the incidence of a specific comorbidity that developed after the index date and its association with OA diagnosed at the index date.

### Data analysis

Each study centre estimated the proportion of each of the specific comorbidities at the index date separately for OA cases and matched controls. We reported centre-specific and pooled proportions and 95% confidence intervals (CI) using the random effect model for congruent evidence. Because the prevalence estimates were similar in scale across centres and not close to 0 or 1, we did not apply a Freeman–Tukey or other variance-stabilising transformation. The pooled estimates were therefore derived directly using random-effects meta-analysis of proportions to preserve interpretability.

In the retrospective analysis, conditional logistic regression was used to calculate both unadjusted and adjusted odds ratios (aOR) of OA for each comorbidity. In the adjusted model, some centres along with age and sex, used different covariates as available in the database. Details of covariates used by different centres are provided in Table 1 and somewhere.^15^ We also calculated the pooled aOR and 95% CI using the random effect model and plotted these using the forest plot.

**Table 1:** Characteristics of the study population across four countries.

	United Kingdom (CPRD-GOLD)		Netherlands (IPCI)		Spain (SIDIAP)		Sweden (SHR)	
	OA	Non-OA	OA	Non-OA	OA	Non-OA	OA	Non-OA
Joint sites included	Hip, Knee, Wrist/Hand, Ankle/Foot, Unspecified		Hip, Knee, Unspecified		Hip, Knee, Wrist/Hand, Ankle/Foot, Generalised, Other		Hip and Knee	
Retrospective analysis								
Study population	259,000	259,000	79,937	318,206	455,494	1,481,298	17,959	17,959
Age at index date in years (mean, SD)	59.40 (12.79)	60.18 (12.82)	65.9 (12.3)	65.8 (12.2)	65.1 (13.6)	64.0 (13.2)	66.7 (11.5)	66.7 (11.5)
Sex, Female (%)	149,523 (57.73)	149,523 (57.73)	51,183 (64.0)	203,747 (64.0)	191,460 (42.03)	617,265 (41.67)	10,698 (59.6)	10,698 (59.6)
BMI (mean, SD)	28.35^a^ (5.67)	26.76^a^ (5.06)	29.0^b^ (5.6)	27.9^b^ (5.9)	NA	NA	NA	NA
Obesity (n, %)	56,106 (21.66)	83,158 (32.11)	7697^b^ (9.62)	18,059^b^ (5.67)	27,870 (6.12)	51,500 (3.48)	NA	NA
Number of conditions studied	57		58		54		37	
Covariates adjusted	Smoking, alcohol, BMI, other comorbidities		age, sex		age, sex		obesity, smoking, alcohol consumption	
Study period	1998–2019		2006–2019		2006–2019		1998–2019	
Prospective analysis								
Study population	259,000	259,000	80,425	1,810,641	455,494	1,481,298	50,942	497,739
Age at index date in years (mean, SD)	59.40 (12.79)	60.18 (12.82)	63.4 (12.3)	42.8 (18.0)	65.1 (13.6)	64.0 (13.2)	65.3 (11.7)	57.3 (14.6)
Sex, Female (%)	149,523 (57.73)	149,523 (57.73)	61.6	50.3	191,460 (42.03)	617,265 (41.67)	29,859 (58.62)	254,593 (51.14)
BMI (mean, SD)	28.35^a^ (5.67)	26.76^a^ (5.06)	NA	NA	NA	NA	NA	NA
Obesity (n, %)	56,106 (21.66)	83,158 (32.11)	NA	NA	27,870 (6.12)	51,500 (3.48)	NA	NA
Number of conditions studied	61		58		57		10	
Covariates adjusted	Age, sex, smoking, alcohol, BMI, other comorbidities		age		age, sex		age, sex, socioeconomic status, birth outside of Sweden, marital status, alcohol, other comorbidities	
Study period	1998–2019		2006–2019		2010–2019		2010–2017	

a N for OA = 249.934; Non-OA = 250,063; NA, Not available.

b Available for 21% of total population.

Except for Sweden, three countries performed prospective analysis for large number of identified conditions. For the prospective analysis, two different methods were used in the four centres according to the suitability of the database and sample sizes. In both the UK and Spain databases participants at risk (i.e., without the specific comorbidity of interest at the index date) were followed up from the index date in parallel for outcomes. Whereas in the Netherlands and Sweden databases a time-varying exposure-controlled cohort design was used, where people aged 18 years or above were followed up. Exposure was defined when a participant developed OA, otherwise non-exposure. Same individual was considered as control till the incidence of OA, and as case, thereafter. Incident comorbidity was counted during the exposure or non-exposure periods within a participant (for those who developed OA), as well as between participants (for those who did not develop OA during the study period). A time-varying exposure analysis was used to compare exposure and non-exposure periods for the incidence of each comorbidity. The Cox regression model was used to calculate unadjusted and adjusted hazard ratios (aHR) for each comorbidity based on the covariates available for each centre (Table 1). The proportional hazards assumption was tested using Schoenfeld residual plots for each analysis. One centre (Sweden) had only eight conditions studied in prospective analysis so we included conditions studied by three or more centres in our pooled estimates.

We reported the congruency of findings across the countries. For illustration and easy interpretation purposes, we presented results of aOR and aHR as follows: i) ‘Congruency’ was present if the results of all the centres favoured one direction (either all significant or all non-significant) ii) ‘Incongruent–all centres did not favour results in the same direction. Centres used the false discovery rate (FDR) or Bonferroni correction method to adjust for multiple testing for comorbidities in their analyses.^23^ Where the associations were congruent, we pooled the aOR and aHR and respective standard errors from all centres and was tested for statistical significance (p < 0.05). Pooled results for incongruent evidence were not emphasised in this manuscript because of nonconsistency, however it is available in Supplementary Files. During the pooled analysis, the heterogeneity between studies was measured using the I^2^ (%) and Q test (P value). For heterogeneity, I^2^ values above 75% were considered as wider heterogeneity, demanding careful interpretation of the findings. We used STATA (SE 17, Texas STATA Corp) and R for the analysis.

### Patient public involvement (PPI)

Three PPI representatives having OA and comorbidities from three different countries, specifically the UK (SV), the Netherlands (JC) and Cyprus (IP) were involved in this study since its inception. They were part of the Project Steering Committee, being involved in the development of the protocol, analysis plan, regular meetings, interpretation and dissemination of the results including newsletters, webpage development, congress abstracts and publications.

### Ethics approval

In the UK, the Clinical Practice Research Data-link (CPRD) contribution was approved by the Independent Scientific Advisory Council (ISAC) 19/30R. In the Netherlands and Spain, the Integrated Primary Care Information (IPCI) registration no. 11/2019 and the Information System for the Development of Research in Primary Care (SIDIAP) 4R19/011 approved it, respectively. In Sweden, the Ethical Review Authority, Skåne Healthcare Register (SHR), ‘Dnr 2011-432, Dnr 2014-276, and Dnr 2018-233, approved it. Each registry database made available anonymised patient records for the research purposes.

### Role of the funding sources

The sponsors did not participate in the design and conduct of the study; collection, management, analysis, and interpretation of the data; or preparation, review, or approval of the manuscript and the decision to submit the manuscript for publication.

## Results

The size of the study population in the four databases varied from 398,143 (Netherlands) to 1,936,792 (Spain), and the number of people with OA varied from 50,942 (Sweden) to 455,494 (Spain). The mean age ranged from 59.8 (SD 12.8) years in UK to 65.8 (SD 12.2) years in the Netherlands. The three databases other than Spain had more women (range 284,452/548,681 = 51.8% in Sweden to 254,930/398,143 = 64% in the Netherlands) than men. Three countries had obesity data recorded which was reported as a prevalence ranging from (79,370/1,936,792) 4.09% in Spain to (139,264/518,000) 26.9% in the UK. The UK and Spain databases had data on hip, knee, wrist/hand, ankle/foot and unspecified OA, whereas the Netherlands included hip, knee and unspecified OA, and Sweden had data only on hip and knee OA (Table 1).

### Prevalence of comorbidities at the index date

**Fig. 1:**
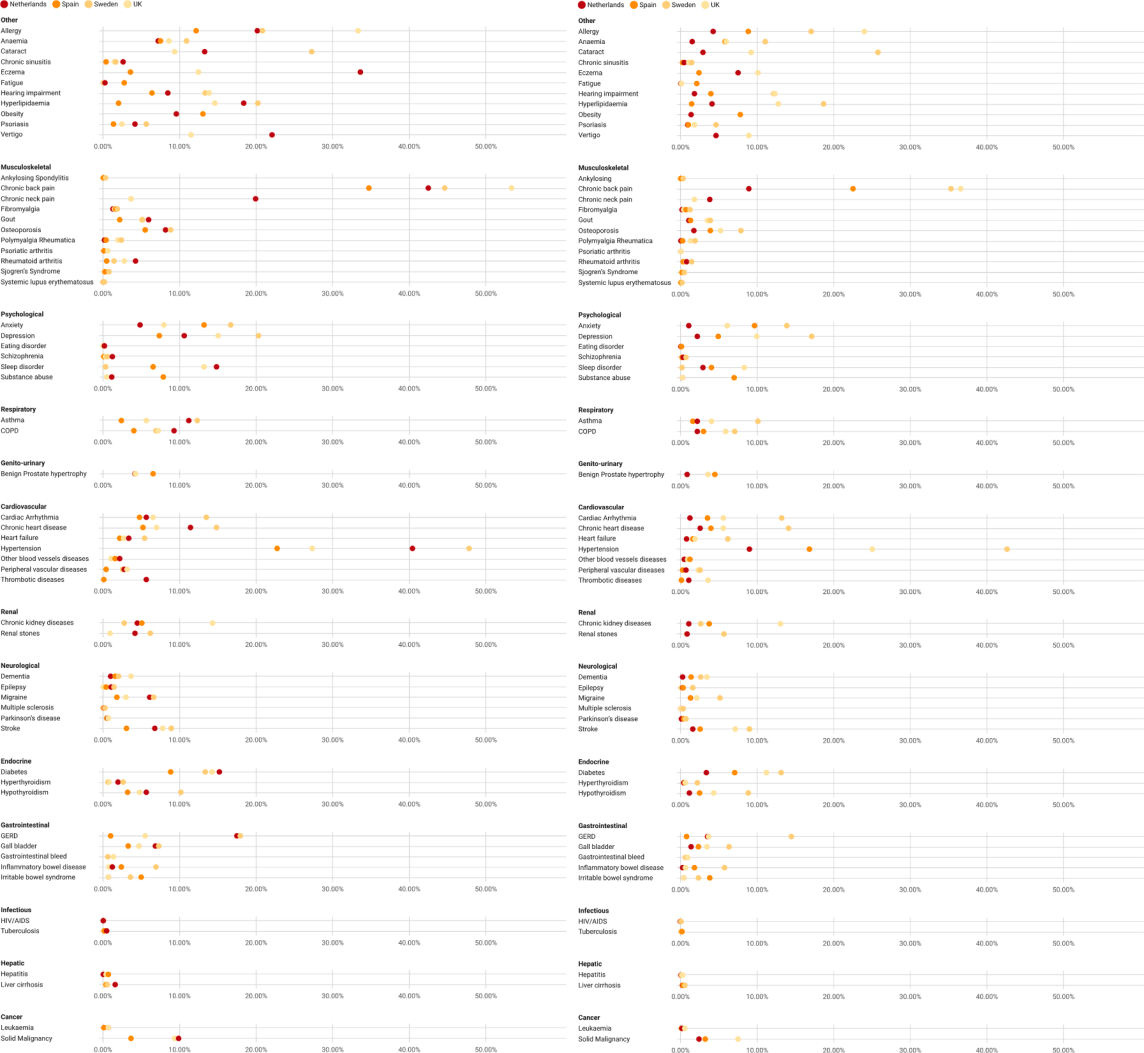
Prevalence of comorbidities at the index date in OA (left) and non-OA (right) across four European countries. GERD, Gastroesophageal reflux disease; COPD, Chronic obstructive pulmonary diseases; IBD, Inflammatory bowel diseases; IBS, irritable bowel syndrome; SLE, Systemic Lupus Erythematous.

In people with OA, the pooled prevalence of comorbidities was highest for chronic back pain (43.8%; 95% CI 33.0–54.8), allergy (21.2%; 95% CI 10.3–34.7), hypertension (34.3%; 95% CI 26.4–42.6), diabetes (12.8%; 95% CI 9.3–16.8), hyperlipidaemia (12.5%; 95% CI 3.6–25.8), depression (13.0%; 95% CI 8.2–18.6), and hearing impairment (HI) (10.3%; 95% CI 6.2–15.3). The non-OA group showed a similar pattern of prevalence but with lower values compared to people with OA (Fig. 1, Supplementary Tables S3 and S4).

### Retrospective analysis

#### Congruent

**Fig. 2:**
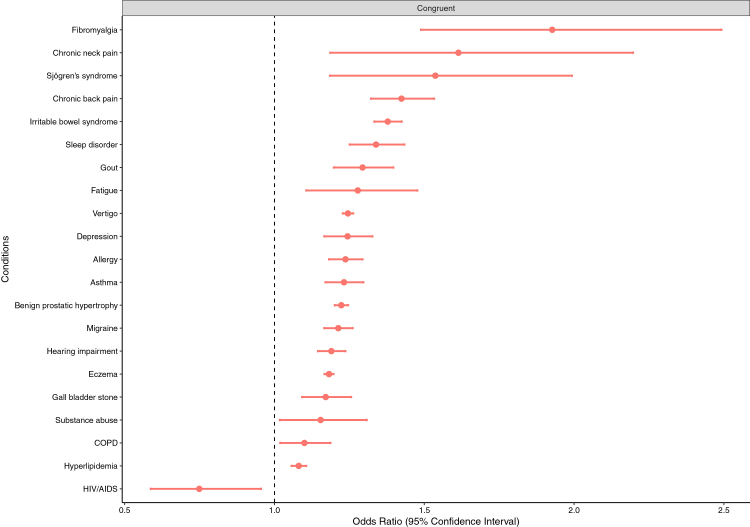
Associations between OA and comorbidities–pooled odds ratio (95% confidence interval) across four European countries (retrospective analysis). COPD, Chronic obstructive pulmonary diseases.

In the retrospective case–control analysis, 21 conditions had congruent evidence of associations with OA. Musculoskeletal (MSK) conditions (e.g. chronic neck pain aOR- 1.6; 95% CI 1.2–2.2, fibromyalgia aOR- 1.9; 95% CI 1.5–2.5) were the leading conditions associated significantly with OA. We also found a significant association of OA with recording of irritable bowel syndrome (IBS) (aOR- 1.4; 95% CI 1.3–1.4), sleep disorder (aOR- 1.3; 95% CI 1.2–1.4), vertigo (aOR- 1.3; 95% CI 1.2–1.4), allergy (aOR- 1.3; 95% CI 1.2–1.4), asthma (aOR- 1.3; 95% CI 1.2–1.4), benign prostatic hypertrophy (BPH) (aOR- 1.3; 95% CI 1.2–1.4), migraine (aOR- 1.3; 95% CI 1.2–1.4), hearing impairment (HI) (aOR- 1.3; 95% CI 1.2–1.4), and eczema (aOR- 1.3; 95% CI 1.2–1.4). (Fig. 2, Supplementary Table S4).

#### Incongruent

A total of 37 conditions did not have congruent evidence of association with OA across four centres. Some of the highlighted conditions are rheumatoid arthritis (RA), GERD, anxiety, hypertension, coronary heart diseases, diabetes, stroke, and dementia (Supplementary Table S5, Supplementary Figure S1).

#### Prospective analysis

**Fig. 3:**
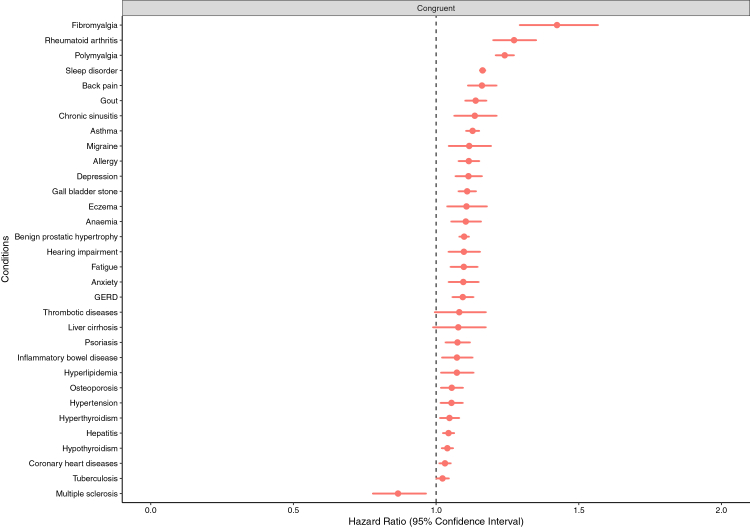
Association between OA and comorbidities–pooled hazard ratio (95% confidence interval) across four European countries (prospective analysis). GERD, Gastroesophageal reflux disease.

In the prospective analysis, of 46 conditions studied by three centres, 32 showed congruent evidence of associations with incident comorbidities with OA. Similar to retrospective findings, MSK (e.g. RA, back pain, fibromyalgia and polymyalgia rheumatica were the related conditions significantly associated with OA (Fig. 3). Other leading conditions which showed congruent and significant evidence for incident comorbidity were chronic sinusitis (aHR- 1.4 95% CI 1.3–1.6), asthma (aHR- 1.3 95% CI 1.2–1.3), migraine (aHR- 1.2 95% CI 1.2–1.3), sleep disorders (aHR- 1.2 95% CI 1.1–1.2), allergy (aHR- 1.2 95% CI 1.1–1.2), eczema (aHR- 1.1 95% CI 1.1–1.2), and BPH (aHR- 1.1 95% CI 1.1–1.2) (Fig. 3). Thrombotic diseases, liver cirrhosis, tuberculosis, and multiple sclerosis had congruent but non-significant associations with OA, whereas incongruent and non-significant evidence was found for 14 comorbidities including diabetes, heart failure, CKD, COPD, and stroke (Supplementary Table S5, Supplementary Figure S2).

Eight conditions were studied by all four centres. The risk of having incident recording for depression (aHR- 1.1 95% CI 1.1–1.2), osteoporosis (aHR- 1.0 95% CI 1.0–1.1), and hypertension (aHR- 1.1 95% CI 1.0–1.1) was higher in people with OA compared to non-OA controls. Five conditions, specifically diabetes, heart failure, COPD, stroke, and dementia were incongruent and had non-significant pooled estimates.

### Concordance of evidence from retrospective and prospective analyses

We compared the ORs and HRs for significant and congruent comorbidities. Fourteen conditions representing 11 different system, were associated both retrospectively and prospectively with OA (Table 2). Beyond musculoskeletal, pain, and cardiovascular conditions the highlight from our study was the association with asthma, migraine, allergy, HI, vertigo, and BPH.

**Table 2:** Conditions having both significant association and congruent in both retrospective and prospective analyses.

Conditions	Prospective HR (95% CI)	Retrospective OR (95% CI)	System
Fibromyalgia	1.42 (1.30–1.57)	1.93 (1.49–2.49)	Pain
Sleep disorder	1.16 (1.15–1.17)	1.34 (1.25–1.43)	Psychological
Chronic back pain	1.16 (1.11–1.21)	1.42 (1.32–1.53)	Musculoskeletal
Gout	1.14 (1.10–1.17)	1.29 (1.19–1.39)	Musculoskeletal
Asthma	1.13 (1.10–1.15)	1.23 (1.17–1.29)	Respiratory
Migraine	1.11 (1.04–1.19)	1.21 (1.17–1.26)	Neurological
Allergy	1.11 (1.08–1.15)	1.24 (1.18–1.29)	Immune
Depression	1.11 (1.07–1.16)	1.24 (1.17–1.33)	Psychological
Gall bladder stone	1.11 (1.08–1.14)	1.17 (1.09–1.26)	Hepatic system
Eczema	1.10 (1.04–1.17)	1.18 (1.17–1.19)	Inflammatory (Skin)
BPH	1.10 (1.08–1.11)	1.22 (1.20–1.25)	Genito-urinary
Hearing impairment	1.10 (1.04–1.15)	1.19 (1.14–1.23)	Auditory
Fatigue	1.09 (1.05–1.15)	1.28 (1.10–1.48)	Musculoskeletal
Hyperlipidaemia	1.07 (1.01–1.13)	1.08 (1.06–1.10)	Cardiovascular

BPH, Benign prostatic hypertrophy; HR, hazard ratio; OR, Odds ratio; CI, Confidence interval.

## Discussion

This is the first large European study on comorbidities in people with OA in primary care settings. The study included 61 conditions and used similar methodology in the four countries to estimate the prevalence and associations of comorbidities before and after the diagnosis of OA. Key findings were:- 1) there was congruent evidence of a higher burden of comorbidities in people with OA at the index date from all four primary care settings; 2) 21 conditions recorded before OA diagnosis, predominantly musculoskeletal (MSK), pain, and sleep disorders, had congruent evidence of associations with OA but no congruent evidence was found towards an association with OA for most of the cardiovascular (CVD) conditions, apart from hypertension; 3) prospectively, 32 out of 46 conditions, (60%) showed congruent and significant associations as an incident comorbidity with OA affecting almost every system of the body; and 4) there was consistent evidence of significant associations with 14 conditions representing multiple systems both before and after the diagnosis of OA by at three centres.

A higher burden of comorbidity in people with OA compared to the non-OA population was evident in all four primary care settings. The prevalence of MSK, psychological, and CVD conditions is similar to that reported in previous studies.^24^, ^25^, ^26^ The wider (>10%) difference in the prevalence for 10 conditions found could be due to variations in country specific primary care practices and guidelines available for diagnosis and recording of the conditions, medication practices and the availability of resources for diagnosis in primary care. For example, under the quality outcome framework (QOF) guideline, general practitioners in the UK get incentives for screening and diagnosing 18 conditions, including CVD.^16^ The country-specific difference in prevalence of certain conditions in people with OA such as pain related conditions in the UK, psychological conditions and hypertension in Sweden, and skin diseases in the Netherlands could reflect a difference in the burden of different chronic conditions in specific country.

Twenty-one conditions, mostly pain related conditions and depression within the group of congruent comorbidities exhibited coherence in evidence from the different countries, despite their differences. As described in previous literature, the relationship of OA with pain and psychological conditions may be explained through bidirectional causal pathways.^27^ The evidence of significant associations of OA with allergy, eczema, HI, vertigo, and benign prostatic hypertrophy (BPH) is an interesting novel finding from our study, which merits further investigation.

The incongruent group of conditions had different results from at least one country. For example, GERD was associated with OA in all countries except Spain, which could be due to different treatment guidelines or recording practices in Spanish primary care. The association with RA is worthy of note, as the association of a previous recording of RA in people with OA was nearly three times higher in the UK, but the no association was found in Spain and Sweden. This perhaps reflects a difference in recording of RA in primary care databases, because RA is one of QOF conditions in the UK NICE guidelines.^28^

A large number of conditions (32 out of the 46 studied) had congruent evidence of associations with OA in the prospective analysis. The list represents different body systems ranging from MSK, to immune and psychological conditions. People diagnosed with OA are especially at higher risk of developing MSK, chronic pain, sleep disorder, and depression conditions in the future.^29^^,^^30^ However, an increased risk of developing allergy and eczema is an interesting result from all four primary care settings. Even though OA is not an immune reactive inflammatory condition, the increased risk of such conditions merits further research.

We found incongruent evidence towards the associations between OA and 14 incident conditions including diabetes, heart failure, and stroke. We did not have information about physical activity and diet in our databases, which is relevant to the development of these conditions and which could vary from country to country.

At least three centres showed consistent significant associations of OA with 14 comorbidities both before and after the index date. People with OA are more at risk of having coexistence of fibromyalgia, chronic back pain, and gout, as reported before.^31^ Similarly, associations of OA with psychological and/or neuro-psychiatric conditions such as depression, insomnia, and migraine may be explained through alterations in central pain mechanisms which associate with sleep problems, fatigue and chronic pain.^32^ The paradox of incongruent association of cardiovascular diseases (CVDs) retrospectively but congruent association of hypertension prospectively may be due to competing risks, as people with CVDs prior to OA are more likely to die. It may be also due to different diagnostic pathways, and surveillance bias in the database and health seeking pattern. This warrants further exploration in datasets with linked mortality and hospitalisation records. Associations with hyperlipidaemia and gall bladder stones might be explained through the increased cholesterol level or obesity of people with OA.^33^ The reporting of HI and BPH with OA is interesting. Prospective associations of OA with HI and BPH might be mediated through ageing despite the use of age-matched controls. No evidence was seen for CVDs in people with OA other than hyperlipidaemia. This could be because of the different control selection or analysis method used in some centres.^34^

We found similar evidence for the burden of comorbidities with OA in four European countries, indicating the presence of MTLCs in OA compared to controls. Associations with a few of the comorbidities have plausible biological explanations but determination of direct or indirect causal relationships for many of the associations needs further study. Concordantly, national guidelines must emphasise the importance of diagnosis and management of MTLCs and understanding of their pattern in people with OA.^35^

Even though we identified 61 conditions to study, the centres could not include all the derived comorbidities because of the lack of codes, availability of the data, validity of the codes, and reliability of the recording of the conditions. Each centre used their available covariates in the analysis. Such heterogeneity may explain partially the difference and similarities in the most of the primary care records used for comparison of the results. We used a reproducibility exercise to standardise the definition of the conditions, code list curation, and covariate selection. There was a different duration of records available for each database, which could have introduced observation period bias. We had inherent limitations of observational data from all the centres and there was no information on over-the-counter prescription of analgesics, physical activity and diet. We could not perform individual patient data analysis because of stringent policy of the data migration of EHRs by different countries. In prospective analysis in Swedish data, the exposure was studied in hip and knee OA only for ten outcomes which needs careful interpretation in pooled results. Misclassification bias for OA and comorbidities is also possible, although most conditions in the study have previously been validated. Therefore, the estimates in our study may not always relate to direct associations between OA and comorbidities but possibly may be mediated through other unrecorded factors as mentioned in the discussion. Although all models were sex-adjusted and controls were matched by sex, we did not perform full sex-stratified analyses. Future studies should explore potential sex-specific differences in OA-associated comorbidities using harmonised datasets with adequate power. However, the primary aim of the study was to compare the associations and distribution of comorbidities in OA rather than to establish causal associations. To some extent the associations may be due to ascertainment bias through increased numbers of visits, especially for the stronger association with OA. Along with possible Berkson's bias, where a physical examination and more investigations are undertaken in people who present with a condition which may detect unsuspected other conditions, a chance of collider bias due to sampling design might also exist. However, we matched the controls having a minimum of 36 months of registration and at least one consultation for any reason. We did not account for competing risks such as all-cause mortality in the survival analyses, which may have led to overestimation of associations in settings where mortality is substantial.

In conclusion, this study provides consistent evidence from four different primary care data in Europe on burden of MLTCs in people living with OA. Association with most of the MLTCs could be explained by shared risk factors, such as altered central pain mechanisms and obesity and many others which still need further investigations. The novel finding of associations with allergy, eczema, HI and BPH provides further scope to explore the mechanistic pathways. The temporal associations reported merit further investigation regarding causality and therapeutic research. The higher burden of MLTCs in people with OA should be considered when developing the shared management plan for this condition.

## Contributors

WZ, MD, CC, SBZ, ME, DA conceived and designed the study. SS, AK, AD AV, and MM developed the methods and performed the analysis, and interpretation of the results. JR, CC, SS, MM accessed and verified the data. CC, WZ, JR, and CFK supervised the statistical analysis. CM, MD, SV, JC, IP guided with clinical and patient oriented interpretations of the results. SS draughted this manuscript and all authors contributed to the critical revision of the manuscript for important intellectual content. JC died, and therefore could not approve the final version of the manuscript. The corresponding author attests that all listed authors meet authorship criteria and that no others meeting the criteria have been omitted.

## Data sharing statement

We used anonymised data on individual patients on which the analysis, results, and conclusions reported in the paper are based. The used data is not distributable under licence. However, the relevant data can be obtained directly from the respective agencies. The codes developed for the analysis can be available upon a valid request to the corresponding author.

## Declaration of interests

WZ declares serving as an advisory board for Ely Lilly (Ixekizumad, 2020) and Regeneron (Fasinomab, 2020). ME declares serving as an advisory Panel Board Member for Pfizer (Nov 2019, Tanezumab). CM provided advice to BMS on recruiting to a non-pharmacological atrial fibrillation trial. The other authors report no financial competing interests. AD is part of the OARSI discussion group on multimorbidity as an unpaid member. SBZ has an Independent Research Grants paid to the Institution from EU, Dutch Arthritis Association and ZonMw, consultant for Pfizer Infirst Healthcare and deputy editor for Osteoarthritis Cartilage journal. The authors are not aware of any conflicts of interest for JC.

## Appendix A Supplementary data

Supplementary File 2

Appendices
