# Supplementary material for: Associations between osteoarthritis and comorbidities before and after diagnosis across four European primary care databases: a multinational case–control and cohort study
Source: Lancet Reg Health Eur. 2026 Aug 11;69:101800. doi: 10.1016/j.lanepe.2026.101800 (PMC13486873; doi:10.1016/j.lanepe.2026.101800)
Supplement: Supplementary file 1 [file mmc1.docx]

**Abstract in different languages**

*This abstract in Swedish, Dutch, Catalan, Spanish, Italian was submitted by the authors and we reproduce it as supplied. It has not been peer reviewed. Our editorial processes have only been applied to the original abstract in English, which should serve as reference for this manuscript.*

1. **Swedish**

**Sammanfattning**

**Bakgrund:** Samsjuklighet vid artros är vanligt, men det råder fortsatt ett flertal oklarheter. Vi analyserade sambanden mellan artros och 61 möjliga komorbiditeter före och efter diagnos med hjälp av data från fyra europeiska primärvårdsdatabaser: Storbritannien, Nederländerna, Sverige och Spanien.

**Metoder**: Multicenterstudien ComOA kombinerade fall-kontroll- och kohortdesigner hos personer i åldern ≥18 år med hjälp av primärvårdsdatabaser från fyra europeiska länder. Studiepopulationen bestod av nya artrosfall och ålders- och könsmatchade kontroller utan artros. Vi rapporterade justerade oddskvoter (aOR) och justerade riskkvoter (aHR) efter justering för relevanta kovariater. Poolade andelar med samsjuklighet, OR och HR uppskattades med hjälp av slumpmässiga effektmetoder för samstämmighet. Samstämmighet förelåg om resultaten från alla fyra länder var likartade.

**Resultat**: De fyra databaserna inkluderade 845 373 artrosfall och 2 556 243 matchade kontroller. Andelen kvinnor varierade från 41,6 % i Spanien till 64,0 % i Nederländerna. Medelåldern vid diagnos av artros var lägst i Storbritannien (59,8 år, SD 12,8) och högst i Nederländerna (65,8 år, SD 12,2). Retrospektivt visade 10 av 33 samsjukligheter som studerades av alla fyra länder samstämmiga samband med artros. De tre vanligaste samsjukligheterna före diagnosen artros var fibromyalgi (aOR 1,93; 95 % KI 1,49–2,49), polymyalgi (aOR 1,44; 95 % KI 1,31–1,58) och kronisk ryggsmärta (aOR 1,42; 95 % KI 1,32–1,53). Prospektivt av åtta tillstånd var sambanden samstämmiga för depression (aHR 1,11; 95 % KI 1,07–1,16), osteoporos (aHR 1,05; 95 % KI 1,01–1,09) och hypertoni (aHR 1,05; 95 % KI 1,02–1,09).

**Tolkning**: Artros är associerat med ett flertal kroniska sjukdomstillstånd i olika länder i Europa, trots olika befolkningsstrukturer och hälsosystem. Ytterligare forskning behövs för att förstå de mekanismer som delas mellan artros och samsjuklighet, särskilt för de komorbiditerer som var samstämmiga mellan de olika länderna.

**Finansiering:** Detta arbete är finansierat av anslag från Foundation for Research in Rheumatology (FOREUM) (anslag 2019-2022), Svenska Vetenskapsrådet (2020-01103), ALF-projektmedel, samt Svenska Reumatikerförbundet. CM är finansierad av National Institute for Health Research (NIHR) Applied Research Collaboration West Midlands, the National Institute for Health Research (NIHR) School for Primary Care Research och National Institute for Health Research (NIHR) Research Professorship in General Practice (NIHR-RP-2014-04-026).

2. **Dutch**

**Achtergrond**
Mensen met artrose hebben vaak ook andere gezondheidsproblemen, zoals pijnklachten, psychische klachten of chronische ziekten. Het is echter niet goed bekend of deze combinaties in verschillende landen even vaak voorkomen. In deze studie onderzochten we de samenhang tussen artrose en andere aandoeningen, zowel vóór als na de diagnose artrose. We gebruikten gegevens uit huisartsenregistraties in vier Europese landen: het Verenigd Koninkrijk, Nederland, Zweden en Spanje.

**Hoe is het onderzoek uitgevoerd?**
We vergeleken volwassenen met een nieuwe diagnose artrose met mensen zonder artrose van dezelfde leeftijd en hetzelfde geslacht. Hiervoor gebruikten we grote databanken uit de eerstelijnszorg (huisartsen). In totaal onderzochten we 61 verschillende aandoeningen. De resultaten uit de vier landen werden samengevoegd en met elkaar vergeleken. We keken vooral naar aandoeningen waarvoor in alle landen een vergelijkbaar verband met artrose werd gevonden.

**Wat vonden we?**
Aan het onderzoek deden ruim 845.000 mensen met artrose mee en meer dan 2,5 miljoen vergelijkbare mensen zonder artrose. De gemiddelde leeftijd waarop artrose werd vastgesteld lag tussen de 60 en 66 jaar.

Bij het terugkijken naar de periode vóór de diagnose artrose bleek dat bij mensen met artrose bepaalde aandoeningen vaker voorkwamen dan bij mensen zonder artrose. Dit gold in alle vier de landen vooral voor fibromyalgie, polymyalgie en chronische rugpijn.

Na de diagnose artrose zagen we dat mensen met artrose iets vaker last kregen van depressie, botontkalking (osteoporose) en hoge bloeddruk. Dit patroon was in alle onderzochte landen vergelijkbaar.

**Wat betekent dit?**
Dit onderzoek laat zien dat artrose in verschillende Europese landen samenhangt met meerdere langdurige aandoeningen, ondanks verschillen in bevolking en zorgsystemen. Dit onderstreept dat artrose niet op zichzelf staat, maar vaak onderdeel is van een breder gezondheidsbeeld.

Meer onderzoek is nodig om beter te begrijpen waarom deze aandoeningen zo vaak samen voorkomen. Die kennis kan helpen bij het verbeteren van de zorg voor mensen met artrose, bijvoorbeeld door meer aandacht te hebben voor andere gezondheidsproblemen die tegelijk kunnen spelen.

3. **Catalan**:

Resum

Antecedents: Les associacions entre osteoartritis (OA) i comorbiditats són freqüents, però es desconeix si es mantenen en diferents sistemes d’atenció primària. Hem analitzat la relació entre OA i 61 comorbiditats abans i després del diagnòstic utilitzant dades de quatre bases europees d’atenció primària: Regne Unit, Països Baixos, Suècia i Espanya.

Mètodes: L’estudi multicèntric ComOA va combinar dissenys d’estudi cas-control i de cohorts en adults ≥18 anys utilitzant grans bases de dades d’atenció primària. La població incloïa casos incidents d’OA i controls sense OA aparellats per edat i sexe. Es va calcular l’odds ratio ajustada (aOR) i el quocient de riscos instantanis ajustat (aHR) ajustant per covariables. Les proporcions combinades de comorbiditats, OR i HR es van estimar amb models d’efectes aleatoris per avaluar la congruència, definida com resultats en la mateixa direcció a tots els centres.

Resultats: Es van incloure 845.373 casos d’OA i 2.556.243 controls. El percentatge de dones va variar del 41,6% a Espanya al 64,0% als Països Baixos. El Regne Unit va presentar la menor edat mitjana de diagnòstic d’OA (59,8 anys; DE 12,8) i els Països Baixos la més alta (65,8 anys; DE 12,2). De les 33 comorbiditats avaluades retrospectivament, 10 van mostrar associacions congruents amb OA a tots els països. Les principals comorbiditats prèvies al diagnòstic van ser fibromiàlgia (aOR 1,93; IC95% 1,49-2,49), polimiàlgia (aOR 1,44; IC95% 1,31-1,58) i dolor lumbar crònic (aOR 1,42; IC95% 1,32-1,53). Prospectivament, les associacions van ser congruents per la depressió (aHR 1,11; IC95% 1,07-1,16), osteoporosi (aHR 1,05; IC95% 1,01-1,09) i hipertensió (aHR 1,05; IC95% 1,02-1,09).

Interpretació: L’OA s’associa amb múltiples malalties cròniques en diferents països europeus, malgrat les diferències entre aquestes poblacions i els seus sistemes sanitaris. Calen més estudis per comprendre els mecanismes compartits entre OA i comorbiditats, especialment en les associacions congruents entre poblacions.

Finançament: Aquest estudi va ser finançat per una beca de la Foundation for Research in Rheumatology (FOREUM, 2019-2022), el Consell Suec d’Investigació (Svenska Vetenskapsrådet, 2020-01103), finançament públic per a recerca clínica al sistema nacional de salut (ALF) i l’Associació Sueca de Reumatisme (Svenska Reumatikerförbundet). CM va rebre finançament del NIHR Applied Research Collaboration West Midlands, del NIHR School for Primary Care Research i del NIHR Research Professorship in General Practice (NIHR-RP-2014-04-026).

4. **Spanish:**

Resumen

Antecedentes: Las asociaciones entre osteoartritis (OA) y comorbilidades son frecuentes. Sin embargo, se desconoce si estas relaciones se repiten de forma similar en diferentes sistemas de atención primaria. Hemos analizado la relación entre OA y 61 comorbilidades antes y después del diagnóstico usando datos de cuatro bases europeas de atención primaria: Reino Unido, Países Bajos, Suecia y España.

Métodos: El estudio multicéntrico ComOA combinó diseños de casos y controles y de cohortes en adultos ≥18 años utilizando grandes bases de datos de atención primaria. La población incluyó casos incidentes de OA y controles sin OA emparejados por edad y sexo. Se calcularon la odds ratio ajustada (aOR) y el cociente de riesgos instantáneos ajustado (aHR) tras ajustar por covariables. Se estimaron las proporciones combinadas de comorbilidades, OR y HR mediante modelos de efectos aleatorios para evaluar la congruencia. Hubo congruencia cuando todos los centros mostraron resultados en la misma dirección.

Resultados: Se incluyeron 845,373 casos de OA y 2,556,243 controles. El porcentaje de mujeres varió desde un 41,6% en España a un 64,0% en los Países Bajos. Reino Unido presentó la menor edad media de diagnóstico de OA (59,8 años; DE 12,8) y Países Bajos la mayor (65,8 años; DE 12,2). Diez de las 33 comorbilidades evaluadas retrospectivamente mostraron asociaciones congruentes con OA en todos los países. Las principales comorbilidades previas al diagnóstico fueron fibromialgia (aOR 1,93; IC95% 1,49-2,49), polimialgia (aOR 1,44; IC95% 1,31-1,58) y dolor lumbar crónico (aOR 1,42; IC95% 1,32-1,53). En el análisis prospectivo, de ocho comorbilidades, las asociaciones fueron congruentes para depresión (aHR 1,11; IC95% 1,07-1,16), osteoporosis (aHR 1,05; IC95% 1,01-1,09) e hipertensión (aHR 1,05; IC95% 1,02-1,09).

Interpretación: La OA está asociada con múltiples enfermedades crónicas en distintos países europeos, a pesar de las diferencias entre estas poblaciones y sus sistemas sanitarios. Se necesitan más estudios para comprender los mecanismos compartidos entre la OA y las comorbilidades, especialmente en las asociaciones congruentes entre poblaciones.

**Financiación:** Este estudio fue financiado por una beca de la Foundation for Research in Rheumatology (FOREUM, 2019-2022), el Consejo Sueco de Investigación (Svenska Vetenskapsrådet, 2020-01103), financiación pública para investigación clínica en el sistema nacional de salud (ALF) y la Asociación Sueca de Reumatismo (Svenska Reumatikerförbundet). CM recibió financiación del National Institute for Health Research (NIHR) Applied Research Collaboration West Midlands, del National Institute for Health Research (NIHR) School for Primary Care Research, y del National Institute for Health Research (NIHR) Research Professorship in General Practice (NIHR-RP-2014-04-026).

5. **Italian**

**Abstract**

**Background:** L’associazione tra l’osteoartrosi (OA) e le comorbidità non sono rare; tuttavia, la consistenza di tali associazioni in diversi contesti di assistenza primaria rimane incerta. Abbiamo analizzato le relazioni tra OA e 61 comorbidità prima e dopo la diagnosi di OA, utilizzando dati provenienti da quattro database europei di assistenza primaria: Regno Unito, Paesi Bassi, Svezia e Spagna.

**Metodi:** Lo studio multicentrico ComOA ha combinato disegni di studio caso-controllo e di coorte in soggetti di età ≥18 anni, utilizzando ampi database di cartelle cliniche elettroniche di assistenza primaria in ciascun Paese. La popolazione in studio era costituita da casi incidenti di OA e da controlli senza OA appaiati per età e sesso. Sono stati riportati odds ratio aggiustati (aOR) e hazard ratio aggiustati (aHR) per altre covariate. Le proporzioni aggregate di comorbidità, così come OR e HR, sono state stimate mediante modelli a effetti casuali per valutare la congruenza delle stime. La congruenza é stata definita come la presenza di stime con la stessa direzione in tutti i centri.

**Risultati:** Tra i quattro database, sono stati inclusi complessivamente 845.373 casi di OA e 2.556.243 controlli appaiati. La percentuale di donne nella popolazione era compresa tra il 41,6% in Spagna e il 64,0% nei Paesi Bassi. L’età media alla diagnosi di OA era più bassa nel Regno Unito (59,8 anni, DS 12,8) e più elevata nei Paesi Bassi (65,8 anni, DS 12,2). Nell’analisi retrospettiva, 10 delle 33 comorbidità studiate in tutti e quattro i Paesi hanno mostrato associazioni con l’OA congruenti. Le tre principali comorbidità precedenti alla diagnosi di OA che hanno mostrato congruenza erano la fibromialgia (aOR 1,93; IC 95% 1,49–2,49), la polimialgia (aOR 1,44; IC 95% 1,31–1,58) e il dolore lombare cronico (aOR 1,42; IC 95% 1,32–1,53). Nell’analisi prospettica, su otto condizioni esaminate, le associazioni erano congruenti per la depressione (aHR 1,11; IC 95% 1,07–1,16), l’osteoporosi (aHR 1,05; IC 95% 1,01–1,09) e l’ipertensione (aHR 1,05; IC 95% 1,02–1,09).

**Interpretazione:** L’OA è associata a molteplici condizioni croniche a lungo termine in diversi Paesi europei, nonostante le differenze nella struttura demografica delle popolazioni e nei sistemi sanitari. Sono necessari ulteriori studi per comprendere i meccanismi condivisi tra OA e comorbidità alla base di queste associazioni, in particolare per quelle risultate congruenti tra popolazioni diverse.

**Finanziamenti:** Questo lavoro è stato sostenuto da un finanizamento della Foundation for Research in Rheumatology (FOREUM) (2019–2022), dal Consiglio Svedese per la Ricerca (2020-01103), da finanziamenti governativi per la ricerca clinica all’interno dei servizi sanitari nazionali (ALF) Svedesi e dalla Associazione di Reumatologia Svedese. CM è finanziato dal National Institute for Health Research (NIHR) Applied Research Collaboration West Midlands, dal NIHR School for Primary Care Research e da una borsa di studio del NIHR per Research Professorship in General Practice (NIHR-RP-2014-04-026).
