# Supplementary material for: Associations between osteoarthritis and comorbidities before and after diagnosis across four European primary care databases: a multinational case–control and cohort study
Source: Lancet Reg Health Eur. 2026 Aug 11;69:101800. doi: 10.1016/j.lanepe.2026.101800 (PMC13486873; doi:10.1016/j.lanepe.2026.101800)
Supplement: Supplementary file 2 [file mmc2.docx]

**Supplementary figure labels**

Supplementary Figure 1. Pooled ORs of conditions with incongruent evidence of association

Supplementary Figure 2. Pooled HRs of conditions with incongruent evidence of association

**Table of content**

[Table 1. List of chronic conditions across four databases	2](#_Toc205552742)

[Table 2. Group of conditions/Outcome	4](#_Toc205552743)

[Table 3. Prevalence of comorbidities in OA across all the centres	5](#_Toc205552744)

[Table 4. Prevalence of comorbidities in non-OA across all the centres	6](#_Toc205552745)

[Table 5. Association of comorbidities with OA preceding the index date across all the centres	7](#_Toc205552746)

[Table 6. I2 values for prospective pooled analysis	10](#_Toc205552747)

[Figure 1. Pooled ORs of conditions with incongruent evidence of association.	8](#_Toc180618742)

[Figure 2. Pooled HRs of conditions with incongruent evidence of association.	9](#_Toc180618743)

Table 1. List of chronic conditions across four databases

| **Sl no** | **Conditions** | **ICD 10** | **ICPC** | **Read Code** |
| --- | --- | --- | --- | --- |
| 1 | Anaemia (All types) | D50-D64 | B78, B80, B81, B82 | D00…, D01… |
| 2 | Ankylosing spondylitis | M45.9 | NA | N10… |
| 3 | Anxiety disorder | F41.0-F41.9 | P74, P74.01, P74.02 | E200… , Eu41… |
| 4 | Asthma | J450-J45.9, J46.9 | R96… | 66Y…, H33… |
| 5 | Benign prostatic hypertrophy (BPH) | N40.9 | Y85 | K20…, K21…, K22… |
| 6 | Cardiac arrhythmias (Atrial Fibrillation) | I47.0-I49.9 | K78 | Gyu… , G573… |
| 7 | Cataract | H25.0-H25.9, H26.1-H26.9 | F92 | F46… , |
| 8 | Chronic Back pain | M47-M48, M51-M54, M99, G54.4 | L02, L03, L86 | N12…, N14…, |
| 9 | Chronic kidney disease ( any cause) | N02.0-N8.8, N11.0-N11.9, N12.9, N15.0-N18.9, N19.9 | U99.01 | 1Z1… , K01…, K02… |
| 10 | Chronic neck pain | M54.2 | L83… | Nyu… , N11…, N12… , N14… |
| 11 | Chronic sinusitis | J32 … | R75… | H13… |
| 12 | Chronic obstructive pulmonary diseases | J41.0-J41.8, J42.9, J43.0-J43.9, J44.0-J44.9 | R91, R95 | H3… |
| 13 | Coronary Heart Disease  (Including Acute Myocardial infarction, Valvular disease, Angina), | I20.0-I25.0, I34.0-I37.0 | K74-K76 | G11…, G30…, G31… - G38.. |
| 14 | Dementia | F00.0-F00.9, F01.0-F03.9, G30.0-G30.9, G31.0-G31.9 | P70-P70.02 | E00…, Eu0…, F11… |
| 15 | Depression | F32.0-F33.9 | P76 | Eu… , E11… |
| 16 | Diabetes mellitus | E10.0-E14.9 | T90…, F83.01 | C10…, F32…, |
| 17 | Dyslipidaemia (Hyper) | E78.1 | T93 | C32… |
| 18 | Eating disorders (Both) |  |  | Eu5… , R03… |
| 19 | Eczema/ Skin disease | L20.0-L22.9, L26.9 | S74, S87, S88 | M11… |
| 20 | Epilepsy | G40.0-G41.9 | N88 | F25… |
| 21 | Fatigue | F48.0 | A04.11 | F286… |
| 22 | Fibromyalgia | M79.7 | L18.01 | N248…, N239… |
| 23 | Gall bladder stone | K80.0-K80.8 | D98-D98.03 | 781…, J65… , 4G2…, |
| 24 | GERD (Gastritis, Oesophageal bleeding, duodenitis, peptic ulcer) | K21 … | D840… | J12… , J13…, J15… |
| 25 | Gastrointestinal bleeding | K25.0-K28.9 | D84-D87 | J11… , |
| 26 | Gout | M10.0-M10.9 | T92 | C34… , N023… |
| 27 | Hearing impairment (All types) | H90.0-H91.9 | H83-H86 | F59… , ZE87… |
| 28 | Heart Failure | 150.0-150.9 | K77-K77.02 | G58…, |
| 29 | Hepatitis | K73.0-K73.9 | D72-D72.05 | J61… , J63… |
| 30 | HIV/AIDS | B20-B24 | B90 | A788… , A789…, AyuC… |
| 31 | Hypertension | I10.9, I11.0-I13.9, I15.0-I15.9 | K86-K87, F83.02 | G20…, G24…, G25…, G26… |
| 32 | Hyperthyroidism | E05.0-E05.9 | T85 | C02… |
| 33 | Hypothyroidism | E02.9, E03.0-E03.9 | T86 | C03…, C04… |
| 34 | Inflammatory Bowel Disease (IBD) | K50.0-K52.9 | D94-D94.02 | J4… , |
| 35 | Irritable Bowel Syndrome (IBS) | K58.1-K58.8 |  | J52… |
| 36 | Leukaemia, Lymphoma | C81.0-C86.6, C91.0-C96.9 | B72-B73 | B60… , B61…, B64… |
| 37 | Liver Cirrhosis | K70.0-K71.9, K74.0-K74.6 | D97… | J615… |
| 38 | Migraine | G43.0-G43.9 | N89 | F26… , |
| 39 | Multiple sclerosis | G35.9 | N86 | F20…, |
| 40 | Osteoarthritis | M16.0-M16.9, M17.0-M17.9 | L89-L91 | N05… , |
| 41 | Osteoporosis | M80.0-M82.9 | L95… | N33… , |
| 42 | Other blood vessel disease (Raynaud’s disease, Burger’s disease) | I73.0-I73.9 | K92… | G73… , |
| 43 | Parkinson’s disease | G20.9 | N87-N87.01 | F12…, |
| 44 | Peripheral vascular disease (Atherosclerosis) | 170.0-170.9 | K91 | G70…, G71… , G72… |
| 45 | Polymyalgia | M35.3 | L99.12 | N20.11 |
| 46 | Psoriasis | L40.0-L41.9 | S91 | M161… |
| 47 | Psoriatic arthritis | M07.0-M07.3 | L99.13 | M160… |
| 48 | Rheumatoid Arthritis | M05.0-M05.9 | L88… , K71… | N04.… |
| 49 | Renal stones | N20.0 …. | U95 | 4G4… , 7B07… , KB12… |
| 50 | Schizophrenia and/or psychosis | F20.0-F20.9, F25.0- F25.9 | P72 | E10… |
| 51 | Severe allergy |  |  | H17… , SN5… |
| 52 | Sjögren's syndrome | M35.0 | NA | N002… |
| 53 | Systemic Lupus Erythematosus | M32.0, M32.1, M32.8, M32.9 | NA | N000… |
| 54 | Sleep disorder (Insomnia) | F51.0 | P06 | Fy0… , 1B1B… |
| 55 | Solid malignancy | C00.0-C80.9, D00.0-D09.9, C97.9 | A29… , A79, B74 – Y78 | B0… - B67… , Byu… |
| 56 | Stroke | G45.0-G46.8, I60.0-I63.9, I65.0-166.9, I69.0-I69.4 | K89-K90.02 | G60… - G68…, F22… |
| 57 | Substance abuse/   Drug addiction | F10.0-F19.9 | P18, P19… | E24…, Eu1… |
| 58 | Thrombotic diseases | I74.0-I74.9 | K93, K94…, W99.03 | G80… , G81… , G74… |
| 59 | Tuberculosis | A15.0-A16.9, B90.9 | A70, R70 | A1…, A11… |
| 60 | Vertigo | H81.4 … | N17-N17.02, H82-H82.03 | R004… , F561… |
| 61 | Vision problem (Glaucoma and other) | H27.0-H27.9, H40.0-H40.9, H42.0-H42.8 | F93…, F94 | F45…, F49… , |

ICD- International Classification of Diseases; ICPC- International classification in primary care. All the codes are the primary code initials used in the database.

Table 2. Group of conditions/Outcome

| Group | Conditions |
| --- | --- |
| Cardiovascular | Cardiac arrhythmias, Coronary Heart Disease (including AMI, valvular disease, angina), Heart failure, Hypertension, Peripheral vascular disease (claudication, Raynaud syndrome, Buerger’s disease), Other blood vessel disease (atherosclerosis and aneurysm), Thrombotic diseases |
| Gastrointestinal | GERD (Esophageal diseases, gastritis, duodenitis), GI bleeding, Inflammatory bowel disease (IBD), Irritable bowel syndrome (IBS) |
| Musculoskeletal | Ankylosing spondylitis, Chronic Back pain, Chronic Neck pain, Fibromyalgia, Polymyalgia, Gout, Osteoporosis, Psoriatic arthritis, Rheumatoid arthritis, Sjögren’s syndrome, Systemic lupus erythematosus (SLE) |
| Endocrine | Diabetes mellitus, Dyslipidaemia (hyper), Hyperthyroidism, Hypothyroidism |
| Neurological | Dementia, Epilepsy, Fatigue, Migraine, Multiple sclerosis, Parkinson disease, Stroke |
| Psychological | Anxiety, Depression, Eating disorders (Anorexia / Bulimia nervosa), Schizophrenia, Sleep disorder (insomnia), |
| Kidney disease | Chronic kidney disease (any cause), Renal stones |
| Liver diseases | Gall bladder stone, Hepatitis, Liver cirrhosis |
| Respiratory | Asthma, Chronic obstructive pulmonary disease (COPD) |
| Cancer | Leukaemia, Lymphoma, Solid Malignancy (any type) |
| Others | Anaemia (all types), Benign prostate hypertrophy (BPH), Cataract, Chronic sinusitis, Eczema/Skin disease, Hearing impairment (all types), Psoriasis, Severe allergy (anaphylactic shock), angioneurotic oedema |

Table 3. Prevalence of comorbidities in OA across all the centres

The pooled prevalence has been estimate using ‘random effect’ model.

|  | Netherlands  (N=79937)  n(%) | Spain  (N=455494)  n(%) | Sweden  (N=17959)  n(%) | UK  (N=259000)  n(%) | Total  (N=812390)  % (95% CI) |
| --- | --- | --- | --- | --- | --- |
| Multimorbidity |  |  |  |  | 39.26 (39.08-39.44) |
| Allergy | 16133 (20.18) | 55552 (12.20) | 3749 (20.88) | 86454 (33.38) | 21.18 (10.31-34.68) |
| Anaemia | 5788 (7.24) | 34349 (7.54) | 1970 (10.97) | 22378 (8.64) | 8.52 (7.63-9.46) |
| Ankylosing | NA | 467 ( 0.10) | 65 ( 0.36) | 130 ( 0.05) | 0.14 (0.07-0.23) |
| Anxiety | 3870 ( 4.84) | 60109 (13.20) | 2993 (16.67) | 20591 ( 7.95) | 10.19 (6.36-14.81) |
| Asthma | 8954 (11.20) | 10992 ( 2.41) | 2215 (12.33) | 14789 ( 5.71) | 7.34 (3.61-12.23) |
| Benign Prostate hypertrophy | 3367 ( 4.21) | 30087 ( 6.61) | NA | 11008 ( 4.25) | 4.97 (3.4-6.82) |
| COPD | 7462 ( 9.33) | 18584 ( 4.08) | 1253 ( 6.98) | 18829 ( 7.27) | 6.79 (4.42-9.62) |
| Cardiac Arrhythmia | 4554 ( 5.70) | 21770 ( 4.78) | 2435 (13.56) | 17094 ( 6.60) | 7.34 (5.59-9.3) |
| Cataract | 10646 (13.32) | NA | 4903 (27.30) | 24372 ( 9.41) | 16.01 (9.62-23.65) |
| Chronic back pain | 34006 (42.54) | 158198 (34.73) | 8022 (44.67) | 138306 (53.40) | 43.78 (33.05-54.82) |
| Chronic kidney diseases | 3587 ( 4.49) | 23150 ( 5.08) | 499 ( 2.78) | 37218 (14.37) | 6.09 (2.2-11.74) |
| Chronic neck pain | 15955 (19.96) | NA | NA | 9531 ( 3.68) | 6.45 (6.37-6.53) |
| Chronic sinusitis | 2099 ( 2.63) | 2067 ( 0.45) | 301 ( 1.68) | 4015 ( 1.55) | 1.46 (0.58-2.72) |
| Chronic heart disease | 9182 (11.49) | 23729 ( 5.21) | 2665 (14.84) | 18104 ( 6.99) | 9.29 (6.48-12.54) |
| Dementia | 844 ( 1.06) | 7306 ( 1.60) | 370 ( 2.06) | 9505 ( 3.67) | 1.99 (0.98-3.36) |
| Depression | 8519 (10.66) | 33604 ( 7.38) | 3656 (20.36) | 39083 (15.09) | 12.98 (8.23-18.63) |
| Diabetes | 12165 (15.22) | 40461 ( 8.88) | 2398 (13.35) | 36985 (14.28) | 12.82 (9.3-16.81) |
| Eating disorder | 170 ( 0.21) | 35 ( 0.19) | NA | 155 ( 0.06) | 0.15 (0.05-0.3) |
| Eczema | 26864 (33.61) | 16420 ( 3.60) | NA | 32271 (12.46) | 14.46 (3.41-31.36) |
| Epilepsy | 907 ( 1.13) | 1935 ( 0.42) | 263 ( 1.46) | 130 ( 0.05) | 0.62 (0.19-1.3) |
| Fatigue | 233 ( 0.29) | 12713 ( 2.79) | 12 ( 0.07) | 466 ( 0.18) | 0.53 (0-2.3) |
| Fibromyalgia | 1046 ( 1.31) | 7696 ( 1.69) | 343 ( 1.91) | 4092 ( 1.58) | 1.6 (1.44-1.77) |
| GERD | 14030 (17.55) | 4845 ( 1.06) | 3231 (17.99) | 14400 ( 5.56) | 8.9 (2.56-18.53) |
| Gall bladder | 5476 ( 6.85) | 15089 ( 3.31) | 1314 ( 7.32) | 12225 ( 4.72) | 5.43 (3.9-7.19) |
| Gastrointestinal bleed | NA | NA | 115 ( 0.64) | 3574 ( 1.38) | 1.32 (1.28-1.37) |
| Gout | 4800 ( 6.00) | 10217 ( 2.24) | 933 ( 5.20) | 13287 ( 5.13) | 4.51 (2.56-6.99) |
| HIV/AIDS | 44 ( 0.06) | 231 ( 0.05) | 8 ( 0.04) | NA | 0.05 (0.04-0.06) |
| Hearing impairment | 6797 ( 8.50) | 29249 ( 6.42) | 2407 (13.40) | 36027 (13.91) | 10.33 (6.24-15.32) |
| Heart failure | 2744 ( 3.43) | 10104 ( 2.22) | 988 ( 5.50) | 7045 ( 2.72) | 3.35 (2.63-4.17) |
| Hepatitis | 328 ( 0.07) | 593 ( 0.74) | NA | 1166 ( 0.45) | NA |
| Hyperlipidaemia | 14743 (18.44) | 9404 ( 2.06) | 3632 (20.22) | 37944 (14.65) | 12.53 (3.61-25.78) |
| Hypertension | 32353 (40.47) | 103788 (22.79) | 8595 (47.86) | 70940 (27.39) | 34.27 (26.44-42.57) |
| Hyperthyroidism | 1567 ( 1.96) | 3369 ( 0.74) | 474 ( 2.64) | 2046 ( 0.79) | 1.42 (0.9-2.06) |
| Hypothyroidism | 4554 ( 5.70) | 14820 ( 3.25) | 1831 (10.20) | 12406 ( 4.79) | 5.73 (4.12-7.59) |
| Inflammatory bowel disease | 990 ( 1.24) | 11150 ( 2.45) | 1247 ( 6.94) | 2098 ( 0.81) | 2.42 (1.17-4.1) |
| Irritable bowel syndrome | 3192 ( 0.70) | 904 ( 5.03) | 9402 ( 3.63) | 3192 ( 0.70) | 2.77 (0.64-6.32) |
| Leukaemia | 577 ( 0.72) | 676 ( 0.15) | NA | 1943 ( 0.75) | 0.49 (0.12-1.12) |
| Liver cirrhosis | 1304 ( 1.63) | 1862 ( 0.41) | 102 ( 0.57) | 958 ( 0.37) | 0.67 (0.32-1.15) |
| Migraine | 4893 ( 6.12) | 8517 ( 1.87) | 1191 ( 6.63) | 7822 ( 3.02) | 4.16 (2.46-6.29) |
| Multiple sclerosis | 159 ( 0.20) | 210 ( 0.05) | 49 ( 0.27) | 285 ( 0.11) | 0.14 (0.07-0.24) |
| Obesity | 7697 ( 9.63) | 59496 (13.06) | NA | NA | 12.52 (12.43-12.61) |
| Osteoporosis | 6555 ( 8.20) | 25123 ( 5.52) | 1588 ( 8.84) | 14271 ( 5.51) | 6.93 (5.78-8.17) |
| Other blood vessels diseases | 1749 ( 2.19) | 7369 ( 1.62) | NA | 2849 ( 1.10) | 1.6 (1.13-2.16) |
| Parkinson’s disease | 514 ( 0.64) | 2445 ( 0.54) | 104 ( 0.58) | 1968 ( 0.76) | 0.63 (0.5-0.77) |
| Peripheral vascular diseases | 2242 ( 2.80) | 2001 ( 0.44) | 465 ( 2.59) | 8133 ( 3.14) | 2.05 (0.54-4.49) |
| Polymyalgia Rheumatica | 185 ( 0.23) | 2110 ( 0.46) | 431 ( 2.40) | 5154 ( 1.99) | 1.06 (0.3-2.27) |
| Psoriasis | 3389 ( 4.24) | 6453 ( 1.42) | 1021 ( 5.69) | 6501 ( 2.51) | 3.26 (1.95-4.88) |
| Psoriatic arthritis | NA | 479 ( 0.11) | NA | 1735 ( 0.67) | 0.25 (0.24-0.27) |
| Renal stones | 3374 ( 4.22) | NA | 1114 ( 6.20) | 2383 ( 0.92) | 3.38 (0.82-7.58) |
| Rheumatoid arthritis | 3398 ( 4.25) | 2297 ( 0.50) | 266 ( 1.48) | 7252 ( 2.80) | 2.01 (0.54-4.39) |
| Schizophrenia | 1020 ( 1.28) | 742 ( 0.16) | 100 ( 0.56) | 958 ( 0.37) | 0.52 (0.19-1.01) |
| Sjogren’s Syndrome | NA | 1286 ( 0.28) | 141 ( 0.79) | 544 ( 0.21) | 0.38 (0.25-0.53) |
| Sleep disorder | 11859 (14.84) | 30016 ( 6.59) | 63 ( 0.35) | 34188 (13.20) | 7.2 (3.3-12.44) |
| Solid Malignancy | 7889 ( 9.87) | 16693 ( 3.66) | NA | 24242 ( 9.36) | 7.34 (3.34-12.73) |
| Stroke | 5452 ( 6.82) | 14193 ( 3.12) | 1607 ( 8.95) | 20357 ( 7.86) | 6.48 (3.59-10.15) |
| Substance abuse | 948 ( 1.19) | 36095 ( 7.92) | NA | 1269 ( 0.49) | 2.38 (0-9.34) |
| Systemic lupus erythematosus | NA | 371 ( 0.08) | 33 ( 0.18) | 414 ( 0.16) | 0.14 (0.07-0.21) |
| Thrombotic diseases | 4541 ( 5.68) | 582 ( 0.13) | NA | 14685 ( 5.67) | 2.94 (0.03-10.26) |
| Tuberculosis | 420 ( 0.53) | 987 ( 0.22) | 25 ( 0.14) | 337 ( 0.13) | 0.23 (0.12-0.38) |
| Vertigo | 17677 (22.11) | NA | NA | 29940 (11.56) | 13.8 (13.68-13.91) |

Table 4. Prevalence of comorbidities in non-OA across all the centres

|  | Netherlands  (N=318206)  n(%) | Spain  (N=1481298)  n(%) | Sweden  (N=17959)  n(%) | UK  (N=259000)  n(%) | Total  (N=2076463)  % (95% CI) |
| --- | --- | --- | --- | --- | --- |
| Multimorbidity |  |  |  |  | 31.07 (30.9-31.24) |
| Allergy | 13727 ( 4.31) | 131131 ( 8.85) | 3062 (17.05) | 62264 (24.04) | 12.56 (5.75-21.54) |
| Anaemia | 5037 ( 1.58) | 86671 ( 5.85) | 1985 (11.05) | 15540 ( 6.00) | 7.11 (6.43-7.83) |
| Ankylosing | NA | 1014 ( 0.07) | 69 ( 0.38) | 26 ( 0.01) | 0.1 (0.03-0.22) |
| Anxiety | 3553 ( 1.12) | 143583 ( 9.69) | 2496 (13.90) | 15799 ( 6.10) | 8.18 (5.2-11.77) |
| Asthma | 7043 ( 2.21) | 24000 ( 1.62) | 1824 (10.16) | 10490 ( 4.05) | 5.6 (2.06-10.74) |
| Benign Prostate hypertrophy | 2746 ( 0.86) | 67034 ( 4.53) | NA | 9428 ( 3.64) | 3.85 (3.13-4.64) |
| COPD | 6951 ( 2.18) | 44371 ( 3.00) | 1272 ( 7.08) | 15281 ( 5.90) | 5.98 (3.11-9.7) |
| Cardiac Arrhythmia | 3959 ( 1.24) | 52036 ( 3.51) | 2374 (13.22) | 14634 ( 5.65) | 6.41 (4.73-8.33) |
| Cataract | 9406 ( 2.96) | NA | 4628 (25.77) | 23906 ( 9.23) | 14.94 (10.77-19.66) |
| Chronic back pain | 28451 ( 8.94) | 334512 (22.58) | 6350 (35.36) | 94768 (36.59) | 32.37 (23.6-41.82) |
| Chronic kidney diseases | 3397 ( 1.07) | 56300 ( 3.80) | 473 ( 2.63) | 33877 (13.08) | 5.39 (2.26-9.75) |
| Chronic neck pain | 12268 ( 3.86) | NA | NA | 4714 ( 1.82) | 7.78 (7.71-7.85) |
| Chronic sinusitis | 1705 ( 0.54) | 4324 ( 0.29) | 263 ( 1.46) | 2901 ( 1.12) | 1.14 (0.31-2.48) |
| Chronic heart disease | 8109 ( 2.55) | 58793 ( 3.97) | 2531 (14.09) | 14504 ( 5.60) | 8.02 (4.72-12.09) |
| Dementia | 1029 ( 0.32) | 20438 ( 1.38) | 482 ( 2.68) | 9013 ( 3.48) | 2.11 (1.23-3.22) |
| Depression | 7013 ( 2.20) | 73584 ( 4.97) | 3082 (17.16) | 25874 ( 9.99) | 9.8 (6.47-13.73) |
| Diabetes | 10843 ( 3.41) | 104945 ( 7.08) | 2364 (13.16) | 29034 (11.21) | 11.11 (7.51-15.31) |
| Eating disorder | 153 ( 0.05) | 25 ( 0.14) | NA | 78 ( 0.03) | 0.11 (0.01-0.28) |
| Eczema | 23945 ( 7.52) | 35776 ( 2.42) | NA | 26211 (10.12) | 11.95 (0.82-33.29) |
| Epilepsy | 855 ( 0.27) | 5579 ( 0.38) | 294 ( 1.64) | 104 ( 0.04) | 0.61 (0.2-1.24) |
| Fatigue | 171 ( 0.05) | 31754 ( 2.14) | 19 ( 0.11) | 285 ( 0.11) | 0.42 (0-1.82) |
| Fibromyalgia | 558 ( 0.18) | 10740 ( 0.73) | 226 ( 1.26) | 1088 ( 0.42) | 0.74 (0.56-0.93) |
| GERD | 11286 ( 3.55) | 11407 ( 0.77) | 2603 (14.49) | 9531 ( 3.68) | 6.82 (1.09-16.89) |
| Gall bladder | 4384 ( 1.38) | 35242 ( 2.38) | 1138 ( 6.34) | 8987 ( 3.47) | 4.27 (2.65-6.25) |
| Gastrointestinal bleed | NA | NA | 124 ( 0.69) | 2383 ( 0.92) | 0.9 (0.87-0.94) |
| Gout | 3498 ( 1.10) | 19994 ( 1.35) | 700 ( 3.90) | 9143 ( 3.53) | 3.16 (1.49-5.42) |
| HIV/AIDS | 63 ( 0.02) | 888 ( 0.06) | 12 ( 0.07) | NA | 0.07 (0.05-0.08) |
| Hearing impairment | 5786 ( 1.82) | 59111 ( 3.99) | 2187 (12.18) | 31935 (12.33) | 8.57 (4.61-13.62) |
| Heart failure | 2478 ( 0.78) | 24259 ( 1.64) | 1121 ( 6.24) | 4999 ( 1.93) | 3 (2.07-4.09) |
| Hepatitis | 901 ( 0.06) | NA | 525 ( 0.16) | 725 ( 0.28) |  |
| Hyperlipidaemia | 13136 ( 4.13) | 21711 ( 1.47) | 3354 (18.68) | 33074 (12.77) | 10.98 (2.4-24.67) |
| Hypertension | 28801 ( 9.05) | 249892 (16.87) | 7664 (42.67) | 65009 (25.10) | 29.66 (19.18-41.35) |
| Hyperthyroidism | 1384 ( 0.43) | 8938 ( 0.60) | 394 ( 2.19) | 1658 ( 0.64) | 1.19 (0.64-1.92) |
| Hypothyroidism | 3780 ( 1.19) | 37628 ( 2.54) | 1592 ( 8.86) | 11318 ( 4.37) | 4.89 (3.3-6.76) |
| Inflammatory bowel disease | 899 ( 0.28) | 27554 ( 1.86) | 1039 ( 5.79) | 1658 ( 0.64) | 1.98 (1.16-3.02) |
| Irritable bowel syndrome | 6580 ( 0.44) | 686 ( 3.82) | 6138 ( 2.37) | 6580 ( 0.44) | 1.93 (0.44-4.43) |
| Leukaemia | 610 ( 0.19) | 2207 ( 0.15) | NA | 1476 ( 0.57) | 0.45 (0.11-1.02) |
| Liver cirrhosis | 944 ( 0.30) | 5452 ( 0.37) | 106 ( 0.59) | 570 ( 0.22) | 0.54 (0.21-1.01) |
| Migraine | 4191 ( 1.32) | 19631 ( 1.33) | 934 ( 5.20) | 5594 ( 2.16) | 3.24 (1.42-5.76) |
| Multiple sclerosis | 241 ( 0.08) | 928 ( 0.06) | 67 ( 0.37) | 259 ( 0.10) | 0.18 (0.07-0.35) |
| Obesity | 4537 ( 1.43) | 115940 ( 7.83) | NA | NA | 7.42 (7.38-7.46) |
| Osteoporosis | 5695 ( 1.79) | 57589 ( 3.89) | 1425 ( 7.93) | 13598 ( 5.25) | 5.94 (4.21-7.95) |
| Other blood vessels diseases | 1562 ( 0.49) | 18481 ( 1.25) | NA | 2435 ( 0.94) | 1.35 (0.92-1.86) |
| Parkinson’s disease | 554 ( 0.17) | 6561 ( 0.44) | 137 ( 0.76) | 1684 ( 0.65) | 0.63 (0.47-0.81) |
| Peripheral vascular diseases | 2246 ( 0.71) | 4386 ( 0.30) | 464 ( 2.58) | 6087 ( 2.35) | 1.8 (0.4-4.17) |
| Polymyalgia Rheumatica | 289 ( 0.09) | 4433 ( 0.30) | 343 ( 1.91) | 3471 ( 1.34) | 0.85 (0.37-1.51) |
| Psoriasis | 2929 ( 0.92) | 15429 ( 1.04) | 830 ( 4.62) | 4817 ( 1.86) | 2.6 (1.26-4.4) |
| Psoriatic arthritis | NA | 1180 ( 0.08) | NA | 233 ( 0.09) | 0.08 (0.08-0.09) |
| Renal stones | 2859 ( 0.90) | NA | 1016 ( 5.66) | 1994 ( 0.77) | 2.95 (0.81-6.38) |
| Rheumatoid arthritis | 2522 ( 0.79) | 5412 ( 0.37) | 265 ( 1.48) | 1839 ( 0.71) | 1.23 (0.25-2.95) |
| Schizophrenia | 1180 ( 0.37) | 2836 ( 0.19) | 126 ( 0.70) | 855 ( 0.33) | 0.59 (0.13-1.37) |
| Sjogren’s Syndrome | NA | 2826 ( 0.19) | 94 ( 0.52) | 285 ( 0.11) | 0.24 (0.14-0.35) |
| Sleep disorder | 9383 ( 2.95) | 60601 ( 4.09) | 35 ( 0.19) | 21678 ( 8.37) | 4.89 (1.87-9.23) |
| Solid Malignancy | 7855 ( 2.47) | 48554 ( 3.28) | NA | 19555 ( 7.55) | 6.6 (2.77-11.9) |
| Stroke | 5174 ( 1.63) | 37789 ( 2.55) | 1613 ( 8.98) | 18467 ( 7.13) | 6.03 (3.17-9.72) |
| Substance abuse | 812 ( 0.26) | 103961 ( 7.02) | NA | 803 ( 0.31) | 1.99 (0-7.97) |
| Systemic lupus erythematosus | NA | 1151 ( 0.08) | 33 ( 0.18) | 181 ( 0.07) | 0.09 (0.07-0.11) |
| Thrombotic diseases | 3422 ( 1.08) | 1681 ( 0.11) | NA | 9454 ( 3.65) | 2.08 (0.03-7.27) |
| Tuberculosis | 381 ( 0.12) | 2773 ( 0.19) | 26 ( 0.14) | 259 ( 0.10) | 0.21 (0.09-0.37) |
| Vertigo | 14844 ( 4.66) | NA | NA | 23155 ( 8.94) | 13.89 (13.8-13.98) |

The pooled prevalence has been estimate using ‘random effect’ model.

Table 5. Association of comorbidities with OA preceding the index date across all the centres

|  | Netherlands | Spain | Sweden | UK | Status | I^2^ |
| --- | --- | --- | --- | --- | --- | --- |
| Allergy | 1.23 ( 1.21 - 1.25 ) | 1.19 ( 1.16 - 1.22 ) | 1.23 ( 1.07 - 1.41 ) | 1.29 ( 1.27 - 1.30 ) | Incongruent | 92.70% |
| Anaemia | 1.16 ( 1.12 - 1.20 ) | 0.99 ( 0.96 - 1.02 ) | 0.95 ( 0.88 - 1.02 ) | 1.40 ( 1.37 - 1.43 ) | Incongruent | 99.10% |
| Ankylosing spondylitis | NA | 1.10 ( 0.84 - 1.44 ) | NA | 2.64 ( 2.12 - 3.28 ) | Incongruent | 95.90% |
| Anxiety | 1.10 ( 1.06 - 1.14 ) | 1.19 ( 1.16 - 1.23 ) | 1.16 ( 0.99 - 1.36 ) | 1.18 ( 1.15 - 1.21 ) | Incongruent | 75.90% |
| Asthma | 1.31 ( 1.28 - 1.34 ) | 1.18 ( 1.14 - 1.22 ) | 1.19 ( 1.1 - 1.27 ) | 1.24 ( 1.21 - 1.27 ) | Congruent | 88.80% |
| Atrial Fibrillation | 1.15 ( 1.11 - 1.19 ) | 1.04 ( 0.99 - 1.09 ) | 1 ( 0.92 - 1.09 ) | 1.22 ( 1.19 - 1.25 ) | Incongruent | 92.80% |
| Benign prostatic hypertrophy | 1.25 ( 1.20 - 1.30 ) | 1.21 ( 1.17 - 1.26 ) | NA | 1.22 ( 1.19 - 1.25 ) | Congruent | 0.00% |
| Cataract | 1.15 ( 1.12 - 1.18 ) | NA | 1.06 ( 0.99 - 1.14 ) | 1.09 ( 1.06 - 1.11 ) | Incongruent | 83.30% |
| Chronic Back pain | 1.34 ( 1.32 - 1.36 ) | 1.55 ( 1.52 - 1.58 ) | 1.45 ( 1.22 - 1.73 ) | 1.38 ( 1.36 - 1.40 ) | Congruent | 97.80% |
| Chronic kidney disease | 1.05 ( 1.01 - 1.09 ) | 0.97 ( 0.94 – 1.03 ) | 1 ( 0.76 - 1.32 ) | 1.06 ( 1.04 - 1.07 ) | Incongruent | 85.30% |
| Chronic neck pain | 1.38 ( 1.35 - 1.41 ) | NA | NA | 1.89 ( 1.82 - 1.97 ) | Congruent | 99.50% |
| Chronic sinusitis | 1.24 ( 1.18 - 1.30 ) | 1.36 ( 1.22 - 1.51 ) | 1.04 ( 0.67 - 1.61 ) | 1.25 ( 1.18 - 1.32 ) | Incongruent | 4.90% |
| COPD | 1.08 ( 1.05 - 1.11 ) | 1.04 ( 1.01 - 1.08 ) | 0.94 ( 0.81 - 1.09 ) | 1.18 ( 1.16 - 1.21 ) | Congruent | 95.50% |
| Coronary Heart Disease | 1.15 ( 1.12 - 1.18 ) | 1.03 ( 0.99 - 1.07 ) | 1.00 ( 0.86 - 1.17 ) | 1.20 ( 1.17 - 1.23 ) | Incongruent | 93.30% |
| Dementia | 0.80 ( 0.74 - 0.86 ) | 0.91 ( 0.85 - 0.97 ) | 0.74 ( 0.64 - 0.86 ) | 1.32 ( 1.28 - 1.36 ) | Incongruent | 98.80% |
| Depression | 1.25 ( 1.22 - 1.28 ) | 1.18 ( 1.14 - 1.22 ) | 1.18 ( 1.04 - 1.32 ) | 1.34 ( 1.31 - 1.37 ) | Incongruent | 93.00% |
| Diabetes | 1.14 ( 1.11 - 1.17 ) | 0.93 ( 0.9 - 0.96 ) | 0.90 ( 0.80 - 1.02 ) | 1.07 ( 1.05 - 1.09 ) | Incongruent | 97.80% |
| Eczema | 1.18 ( 1.16 - 1.20 ) | 1.20 ( 1.15 - 1.25 ) | NA | 1.18 ( 1.16 - 1.21 ) | Congruent | 0.00% |
| Epilepsy | 1.06 ( 0.98 - 1.14 ) | 0.97 ( 0.86 - 1.10 ) | 0.90 ( 0.71 - 1.14 ) | 1.16 ( 1.06 - 1.27 ) | Incongruent | 60.60% |
| Fatigue | 1.37 ( 1.18 - 1.59 ) | 1.15 ( 1.10 - 1.20 ) | NA | 1.39 ( 1.18 - 1.63 ) | Congruent | 78.10% |
| Fibromyalgia | 1.91 ( 1.77 - 2.06 ) | 1.69 ( 1.58 - 1.81 ) | 1.27 ( 0.76 - 2.12 ) | 2.70 ( 2.51 - 2.91 ) | Congruent | 96.70% |
| Gall bladder stone | 1.27 ( 1.23 - 1.31 ) | 1.08 ( 1.03 - 1.13 ) | 1.13 ( 1.03 - 1.23 ) | 1.20 ( 1.17 - 1.24 ) | Congruent | 91.30% |
| GERD | 1.29 ( 1.26 - 1.32 ) | 1.02 ( 0.94 - 1.11 ) | 1.25 ( 1.10 - 1.41 ) | 1.39 ( 1.35 - 1.43 ) | Incongruent | 94.50% |
| Gout | 1.40 ( 1.35 - 1.45 ) | 1.18 ( 1.12 - 1.24 ) | 1.25 ( 1.03 - 1.51 ) | 1.32 ( 1.28 - 1.36 ) | Congruent | 90.10% |
| Hearing impairment | 1.19 ( 1.16 - 1.23 ) | 1.26 ( 1.22 - 1.30 ) | 1.09 ( 1.01 - 1.18 ) | 1.18 ( 1.16 - 1.20 ) | Congruent | 83.80% |
| Heart Failure | 1.10 ( 1.05 - 1.15 ) | 0.91 ( 0.86 - 0.96 ) | 0.80 ( 0.71 - 0.89 ) | 1.37 ( 1.32 - 1.43 ) | Incongruent | 98.40% |
| Hepatitis | 1.14 ( 1.04 - 1.25 ) | 1.12 ( 0.82 - 1.53 ) | NA | 1.39 ( 1.25 - 1.54 ) | Incongruent | 75.70% |
| HIV/AIDS | 0.71 ( 0.51 - 0.97 ) | 0.81 ( 0.56 - 1.18 ) | NA | NA | Congruent | 0% |
| Hyperlipidemia | NA | 1.05 ( 1.01 - 1.11 ) | 1.04 ( 0.92 - 1.18 ) | 1.09 ( 1.07 - 1.11 ) | Congruent | 10.40% |
| Hypertension | 1.22 ( 1.20 - 1.24 ) | 1.01 ( 0.99 - 1.03 ) | 1.20 ( 1.12 - 1.29 ) | 1.04 ( 1.02 - 1.05 ) | Incongruent | 98.90% |
| Hyperthyroidism | 1.13 ( 1.07 - 1.20 ) | 0.92 ( 0.84 - 1.01 ) | 1.18 ( 1.02 - 1.38 ) | 1.04 ( 0.89 - 1.21 ) | Incongruent | 80.90% |
| Hypothyroidism | 1.22 ( 1.18 - 1.26 ) | 0.99 ( 0.95 - 1.04 ) | 1.12 ( 1.03 - 1.21 ) | 1.04 ( 1.01 - 1.07 ) | Incongruent | 95.70% |
| Inflammatory bowel disease | 1.10 ( 1.02 - 1.18 ) | 1.06 ( 1.01 - 1.11 ) | 1.14 ( 0.96 - 1.36 ) | 1.17 ( 1.08 - 1.26 ) | Congruent | 39.30% |
| Irritable bowel syndrome | NA | 1.39 ( 1.26 - 1.53 ) | 1.27 ( 1.02 - 1.58 ) | 1.38 ( 1.33 - 1.43 ) | Incongruent | 0.00% |
| Leukaemia, Lymphoma | 0.94 ( 0.86 - 1.03 ) | 0.91 ( 0.76 - 1.09 ) | 0.96 ( 0.78 - 1.18 ) | 1.34 ( 1.24 - 1.45 ) | Incongruent | 92.80% |
| Liver Cirrhosis | 1.39 ( 1.30 - 1.48 ) | 0.99 ( 0.88 - 1.12 ) | NA | 1.35 ( 1.20 - 1.51 ) | Incongruent | 91.80% |
| Migraine | 1.19 ( 1.15 - 1.23 ) | 1.17 ( 1.12 - 1.24 ) | 1.27 ( 1.10 - 1.46 ) | 1.26 ( 1.21 - 1.31 ) | Congruent | 54.10% |
| Multiple sclerosis | 0.66 ( 0.56 - 0.78 ) | 0.74 ( 0.52 - 1.06 ) | NA | 0.96 ( 0.79 - 1.16 ) | Incongruent | 75.50% |
| Osteoporosis | 1.16 ( 1.13 - 1.20 ) | 1.13 ( 1.06 - 1.15 ) | 1.09 ( 0.83 - 1.44 ) | 1.18 ( 1.15 - 1.21 ) | Incongruent | 63.80% |
| Other vessel diseases | 1.12 ( 1.06 - 1.18 ) | 1.04 ( 0.98 - 1.1 ) | NA | 1.21 ( 1.14 - 1.29 ) | Incongruent | 83.80% |
| Parkinsonian disease | 0.92 ( 0.83 - 1.01 ) | 0.87 ( 0.77 - 0.99 ) | NA | 1.25 ( 1.16 - 1.34 ) | Incongruent | 94.80% |
| Peripheral vascular disease | 0.99 ( 0.94 - 1.04 ) | 1.22 ( 1.08 - 1.33 ) | 1.00 ( 0.74 - 1.36 ) | 1.32 ( 1.27 - 1.37 ) | Incongruent | 96.60% |
| Polymyalgia rheumatica | 1.46 ( 1.31 - 1.63 ) | 1.32 ( 1.16 - 1.45 ) | 1.27 ( 0.78 - 2.05 ) | 1.54 ( 1.47 - 1.61 ) | Incongruent | 63.80% |
| Psoriasis | 1.16 ( 1.12 - 1.21 ) | 1.11 ( 1.04 - 1.19 ) | 1.19 ( 1.00 - 1.41 ) | 1.24 ( 1.19 - 1.29 ) | Incongruent | 68.80% |
| Psoriatic Arthritis | NA | 0.95 ( 0.76 - 1.19 ) | NA | 4.01 ( 3.19 - 4.83 ) | Incongruent | 99.50% |
| Renal stones | 1.19 ( 1.14 - 1.24 ) | NA | 1.06 ( 0.97 - 1.17 ) | 1.09 ( 1.02 - 1.16 ) | Incongruent | 75.80% |
| Rheumatoid Arthritis | 1.36 ( 1.31 - 1.42 ) | 1.04 ( 0.91 - 1.18 ) | 0.76 ( 0.57 - 1.02 ) | 3.72 ( 3.51 - 3.94 ) | Incongruent | 99.70% |
| Schizophrenia | 0.86 ( 0.80 - 0.92 ) | 0.68 ( 0.55 - 0.84 ) | NA | 0.97 ( 0.87 - 1.08 ) | Incongruent | 78.10% |
| Sjogren's syndrome | NA | 1.35 ( 1.17 - 1.55 ) | NA | 1.76 ( 1.50 - 2.07 ) | Congruent | 83.10% |
| Sleep disorder (Insomnia) | 1.31 ( 1.28 - 1.34 ) | 1.28 ( 1.24 - 1.32 ) | NA | 1.43 ( 1.40 - 1.46 ) | Congruent | 95.70% |
| Solid malignancy | 1.01 ( 0.97 - 1.03 ) | 0.98 ( 0.94 - 1.02 ) | 0.96 ( 0.91 - 1.02 ) | 1.27 ( 1.25 - 1.29 ) | Incongruent | 99.00% |
| Stroke | 1.19 ( 1.15 - 1.23 ) | 0.95 ( 0.91 - 1 .03) | 0.98 ( 0.84 - 1.13 ) | 1.13 ( 1.10 - 1.16 ) | Incongruent | 95.30% |
| Substance abuse | 1.17 ( 1.09 - 1.26 ) | 1.04 ( 1.01 - 1.07 ) | NA | 1.29 ( 1.17 - 1.43 ) | Congruent | 90.70% |
| Systemic Lupus Erythematosus | NA | 0.92 ( 0.71 - 1.23 ) | NA | 1.76 ( 1.46 - 2.13 ) | Incongruent | 93.40% |
| Thrombotic diseases | 1.34 ( 1.29 - 1.39 ) | 0.95 ( 0.75 - 1.22 ) | NA | 1.46 ( 1.42 - 1.51 ) | Incongruent | 91.60% |
| Tuberculosis | 1.10 ( 0.99 - 1.23 ) | 1.07 ( 0.87 - 1.31 ) | NA | 1.21 ( 1.01 - 1.44 ) | Incongruent | 0.00% |
| Vertigo | 1.25 ( 1.23 - 1.27 ) | NA | NA | 1.24 ( 1.22 - 1.26 ) | Congruent | 0.00% |
| Vision problem | 1.07 ( 1.03 - 1.12 ) | NA | 1.07 ( 0.98 - 1.16 ) | 1.04 ( 1.01 - 1.07 ) | Incongruent | 0.00% |

COPD- Chronic Obstructive Pulmonary Diseases, GERD- Gastro Esophageal Reflux Disorder

Figure 1. Pooled ORs of conditions with incongruent evidence of association.

Figure 2. Pooled HRs of conditions with incongruent evidence of association.

Table 6. I2 values for prospective pooled analysis

| Conditions | I^2^ |
| --- | --- |
| Allergy | 95.46 |
| Anaemia | 95.35 |
| Ankylosing spondylitis | 56.01 |
| Anxiety | 97.43 |
| Asthma | 74.92 |
| Atrial Fibrillation | NA |
| Benign prostatic hypertrophy | 45.89 |
| Cataract | 99.08 |
| Chronic Back pain | 98.51 |
| Chronic kidney disease | 97.99 |
| Chronic neck pain | 97.53 |
| Chronic sinusitis | 82.04 |
| COPD | 98.45 |
| Coronary Heart Disease | 49.39 |
| Dementia | 99.35 |
| Depression | 96.11 |
| Diabetes | 98.55 |
| Eczema | 98.1 |
| Epilepsy | 56.96 |
| Fatigue | 39.8 |
| Fibromyalgia | 82.63 |
| Gall bladder stone | 86.17 |
| GERD | 87.91 |
| Gout | 80.26 |
| Hearing impairment | 96 |
| Heart Failure | 97.26 |
| Hepatitis | NA |
| Hyperlipidemia | 96.9 |
| Hypertension | 97.78 |
| Hyperthyroidism | 33.72 |
| Hypothyroidism | 76.97 |
| Inflammatory bowel disease | 84.63 |
| Irritable bowel syndrome | 39.1 |
| Leukaemia, Lymphoma | 69.28 |
| Liver Cirrhosis | 80.01 |
| Migraine | 94.1 |
| Multiple sclerosis | NA |
| Osteoporosis | 88.3 |
| Other vessel diseases | 76.71 |
| Parkinsonian disease | 65.51 |
| Peripheral vascular disease | 95.76 |
| Polymyalgia rheumatica | NA |
| Psoriasis | 78.18 |
| Psoriatic Arthritis | NA |
| Renal stones | 0.03 |
| Rheumatoid Arthritis | 66.72 |
| Schizophrenia | 94.52 |
| Sjogren's syndrome | NA |
| Sleep disorder (Insomnia) | 96.55 |
| Solid malignancy | 85.07 |
| Stroke | 92.81 |
| Substance abuse | 27.52 |
| Systemic Lupus Erythematosus | 93.6 |
| Thrombotic diseases | NA |
| Tuberculosis | 0.16 |
| Vertigo | 74.73 |
| Vision problem | 0.03 |
